# Active zone plasticity couples sleep need to presynaptic hypophosphorylation

**DOI:** 10.1073/pnas.2524065123

**Published:** 2026-06-08

**Authors:** Chengji Piao, Ewelina P. Dutkiewicz, Laxmikanth Kollipara, Albert Sickmann, Sheng Huang, Stephan J. Sigrist

**Affiliations:** ^a^ Institute for Biology/Genetics, Freie Universität Berlin, Berlin 14195, Germany; ^b^NeuroCure Cluster of Excellence, Charité Universitätsmedizin, Berlin 10117, Germany; ^c^Leibniz-Institut für Analytische Wissenschaften, Dortmund 44139, Germany; ^d^ Medizinische Fakultät, Ruhr-Universität Bochum, Bochum 44801, Germany

**Keywords:** Bruchpilot (BRP), active zone, synaptic plasticity, phosphorylation, sleep homeostasis

## Abstract

Sleep need is associated with both circuit dynamics and widespread synaptic plasticity, yet the specific synaptic changes underlying sleep homeostasis remain incompletely understood. In *Drosophila*, sleep loss has been shown to trigger plasticity of the presynaptic active zone, marked by increasing levels of the ELKS-family scaffold protein Bruchpilot (BRP). By titrating *brp* gene copy number, we previously established a presynapse-specific, dosage-dependent paradigm that modulates sleep pressure. Here, to elucidate the molecular landscape of this plasticity, we performed synapse-enriched integrated-omics. Proteomic and bioinformatic analyses revealed changes in immune and stress response pathways and local translation control. Strikingly, phospho-proteomic analysis uncovered a global shift toward hypophosphorylation, particularly in presynaptic proteins, indicating a reprogramming of the phosphorylation–dephosphorylation balance. This presynaptic hypophosphorylation is likely contributed by reduced activity of Protein Kinase A (PKA) and enhanced substrate affinity of Protein Phosphatase 1 (PP1) mediated by its regulatory subunit Spinophilin (Spn). Manipulating either PKA or PP1 activity was sufficient to suppress BRP-modulated sleep phenotypes. We propose that presynaptic hypophosphorylation constitutes a molecular signature of local synaptic remodeling that adaptively tunes sleep need via reversible posttranslational modification, a mechanism likely conserved across species.

## Significance

Synaptic plasticity has been causally linked to homeostatic sleep regulation. However, how synaptic plasticity governs the dynamic accumulation and dissipation of sleep pressure remains elusive. Using synapse-enriched proteomics and phospho-proteomics, we found that sleep pressure is associated with a coordinated regulation of phosphorylation confined to the presynaptic compartment, while postsynaptic regions remain largely unaffected. This phospho-imbalance is likely maintained by modulating kinase and phosphatase activities, including reduced phosphorylation of PKA at its activating site and enhanced targeting of PP1 via hypophosphorylated Spinophilin. Such a presynapse-restricted hypophosphorylation scenario emerges as a core feature of sleep pressure and suggests an evolutionarily conserved molecular signature and spatial phospho-distribution for maintaining sleep homeostasis.

Sleep is a conserved animal behavior thought to serve fundamental physiological functions, as highlighted by the fact that we spend roughly one-third of our lives asleep and suffer severe consequences from sleep loss (1, 2). The brain, as a central organ in sleep regulation, relies on precise synaptic transmission, particularly the fusion of neurotransmitter-filled synaptic vesicles (SVs) at specialized presynaptic release structures known as the active zone (3). Although the fundamental functions of sleep remain under intensive investigation, and various factors have been implicated in its regulation (4), a growing body of work has linked sleep to synaptic plasticity across species (5–10). According to the synaptic homeostasis hypothesis, sleep renormalizes synaptic gains accumulated during wake phases and reduces the burden of saturated plasticity (11, 12), a view supported by structural and proteomic evidence of synapse scaling during sleep–wake cycle (5–8, 10, 13). Beyond structural remodeling, dynamic oscillation of synaptic phospho-proteome driven by sleep–wake cycle has been observed in the mouse forebrain (14). Moreover, several kinases have been implicated in sleep regulation in both flies and mammals (15–20). More recently, phosphatases such as PP1 and calcineurin (PP2B) have been shown to counterbalance kinase signaling at postsynapse to regulate sleep (21, 22). While protein phosphorylation is critical for the regulation of synapse function and plasticity, how phosphorylation dynamics are coordinated with structural synaptic plasticity to tune synaptic transmission and sleep behavior remains unclear, particularly from a presynaptic perspective, wherein the release site number and release probability are important factors determining synaptic strength and plasticity.

In *Drosophila*, previous work established that presynaptic plasticity driven by the ELKS-family core active zone scaffold protein Bruchpilot (BRP) acts as a direct actuator of sleep homeostasis (6, 8, 9, 23–27). Such a remodeling of the presynaptic release machinery driven by BRP provokes sleep in a dosage-dependent manner and reprograms the neuronal activity in the sleep pressure circuity to regulate sleep (8, 9, 25, 28). Furthermore, it also promotes adaptive sleep pattern changes and mediates a tradeoff between memory and longevity during early brain aging (9, 29). In this study, we employed this genetic model of high sleep need in combination with global proteomic and phospho-proteomic analyses to define the molecular landscape by which BRP-dependent presynaptic plasticity links synaptic remodeling to sleep homeostasis. Specifically, we dissect how structural plasticity at presynaptic active zones coordinates local phosphorylation signaling to modulate synaptic function and regulate sleep.

## Results

### Proteomic and Phospho-Proteomic Profiling of BRP-Dependent Presynaptic Plasticity.

**Fig. 1.**
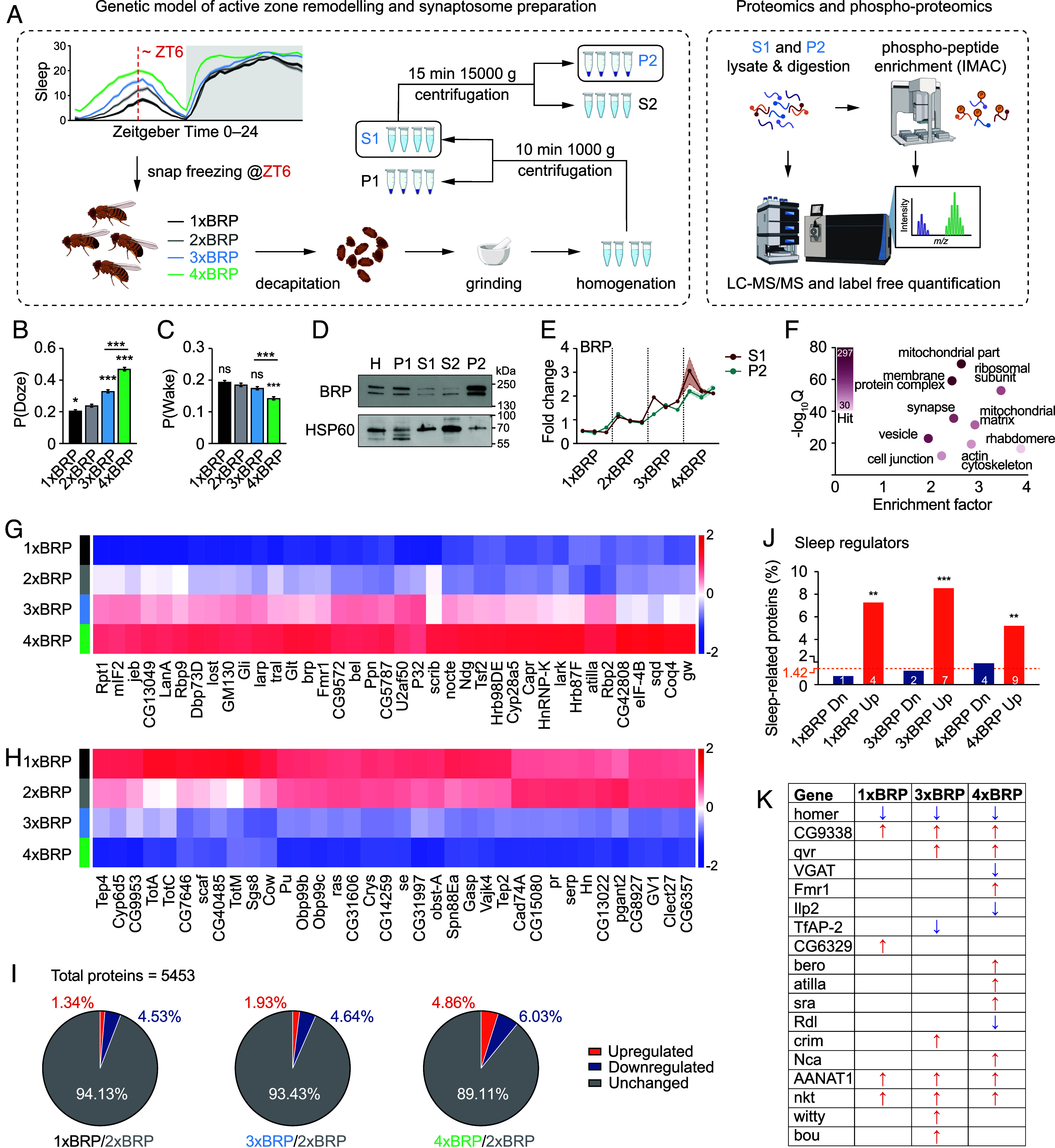
Synaptosomal proteomic characterization of BRP-dependent presynaptic plasticity. (*A*) Experiment and bioinformatic analysis workflow of the proteomic and phospho-proteomic investigation of BRP-dependent presynaptic plasticity. (*B* and *C*) Sleep structure of 1 to 4xBRP flies including sleep pressure shown by P(Doze) (*B*) and sleep depth shown by P(Wake) (*C*). One-way ANOVA with Tukey’s post hoc tests. n = 127 to 128. (*D*) Western blotting of BRP as a marker of synaptic component and Hsp60 as a marker of the mitochondrial component in different biochemical fractions of synaptosome preparation. H, head homogenate; P1, head homogenate pellet; S1, head homogenate supernatant; S2, soluble fraction enriched for microsomes and SVs; P2, synaptosome fraction. (*E*) BRP levels across three independent biological samples in 1 to 4xBRP animals. (*F*) Significantly enriched cellular components in the synaptosome P2 fraction. Significance is plotted against the enrichment factor of each cellular component. The color gradient of dots represents the number of proteins detected in the category. (*G* and *H*) Proteins scaled up (*G*) or down (*H*) with BRP in the synaptosome P2 fraction. The heat maps show the normalized Z-score. (*I*) Distribution of proteins with significantly upregulated and downregulated levels in the synaptosome of 1xBRP, 3xBRP, and 4xBRP compared to 2xBRP control. A total of 5,453 proteins were detected in the synaptosome P2 fraction across different samples. (*J* and *K*) Percentages of sleep-related proteins among upregulated and downregulated proteins when comparing 1xBRP, 3xBRP, or 4xBRP to 2xBRP control in the synaptosome P2 fraction. The numbers of sleep-related proteins are indicated (*J*). Sleep-related proteins showing significant differences in each pairwise comparison are listed in (*K*). Fisher’s exact test, two-sided. *P < 0.05; **P < 0.01; ***P < 0.001; ns, not significant. Error bars: mean ± SEM. ↑ upregulation, ↓ downregulation.

To deepen our understanding of how BRP-dependent presynaptic plasticity executes sleep homeostasis, we intended to characterize its global molecular landscape. BRP-dependent presynaptic plasticity was genetically induced by adjusting the *brp* gene copy numbers from one to four, generating 1xBRP, 2xBRP, 3xBRP, and 4xBRP flies. 2xBRP corresponds to the *wild-type* (*wt*) background control, and all lines were backcrossed to this genetic background to ensure isogeneity. To collect samples for omics analysis, 5-d-old flies, which normally show mature sleep behavior, were snap-frozen in liquid nitrogen at the middle of daytime (zeitgeber time 6, ZT6), when the sleep differences from one to four *brp* copies are most profound (Fig. 1*A*) (8). We also revisited the sleep structure of these flies and confirmed that BRP-dependent presynaptic plasticity dictated sleep by controlling the sleep pressure in a BRP dosage-dependent manner, indicated by a near-linear increase of P(Doze) (Fig. 1*B*), while a linear tendency of higher sleep quality demonstrated by lower P(Wake) (30) could be observed as well (Fig. 1*C*). We then performed synaptosome preparations from fly heads to biochemically enrich synaptic materials. Through differential centrifugation (Fig. 1*A*), we obtained three fractions for each genetic group: 1) the head homogenate supernatant S1 fraction; 2) the synaptosome P2 fraction; and 3) the soluble S2 fraction which is enriched for microsomes and SVs (31). The synaptosome preparation procedure effectively enriched synaptic proteins as exemplified by an enrichment of BRP in the P2 fraction (Fig. 1*D*).

To obtain a complete overview on both the global and synapse-specific proteome changes, we subject the head homogenate S1 fraction and the synaptosome P2 fraction to quantitative label-free mass spectrometry for assessing both protein abundance and the protein phosphorylation level (Fig. 1*A*). Abundances of all identified BRP isoforms in both fractions exhibited a near-linear increase from one to four *brp* gene copies (Fig. 1*E*). This quantitative proteomic analysis of BRP protein abundance is consistent with our previous western blot analysis (8), supporting that genetic presynaptic plasticity induction by tuning *brp* gene copies from one to four triggers a dynamic range of BRP protein levels comparable to that reported previously in short-sleeping mutants (8, 9, 26). Global proteomics identified 5,453 protein isoforms corresponding to 2,967 unique proteins in the synaptosome P2 fraction (*SI Appendix*, Fig. S1 *A* and *B*). To assess the quality of the fractionation, we performed a Gene Ontology (GO) enrichment analysis for cellular components on the synaptosome dataset. The terms including synapse, cell junction, vesicle, and mitochondrial components ranked among the top enriched categories (Fig. 1*F*), demonstrating an effective enrichment of the synaptic materials through synaptosome preparation.

### Synapse-Localized Protein Abundance Changes Associated with BRP Levels.

Following synaptosome fractionation and mass spectrometric quantification, we performed an in-depth bioinformatic analysis of these datasets. Given that BRP is a core presynaptic scaffold which drives presynaptic active zone remodeling and promotes sleep in a dosage-dependent manner (6, 8, 9, 23–26), we started by searching for proteins whose abundance profiles followed that of BRP in the synaptosome P2 fraction. Among these, we identified a distinct cluster of RNA binding proteins involved in translation control and mRNA splicing (U2af50, sqd, Hrb98DE, HnRNP-K, Hrb87F, Rbp2, and Rbp9), as well as a group of proteins that play a role in the regulation of circadian and locomotor rhythms: Gawky (32), Lark (33, 34), and Nocte (35) (Fig. 1*G*). Importantly, this group also includes Fmr1 (Fig. 1 *G* and *K*), which has been shown to bind mRNAs encoding approximately one-third of all the presynaptic proteins (36) and found to be required presynaptically for structural and functional presynaptic plasticity (37). Interestingly, although all of the proteins mentioned above were also detected in the head homogenate supernatant S1 fraction, they did not show the same BRP-correlated scaling-up pattern. This suggests that their regulation is locally confined to synaptic regions rather than occurring uniformly across the neuron. Meanwhile, members of the Turandot family proteins, which are typically induced in response to severe stress (38, 39), were found to be negatively correlated with BRP levels (Fig. 1*H*). Turandot proteins are also involved in immune response, and Turandot A in particular serves as a marker of JAK/STAT pathway activation (40). In addition, complement-like thioester-containing proteins TEP2 and TEP4, whose expression is induced by immune challenge via the JAK/STAT pathway (41), were also found to be negatively correlated with BRP levels (Fig. 1*H*). Notably, the JAK/STAT pathway has been found involved in long-term synaptic plasticity (42) and is known to cycle under the control of the circadian clock (43), further connecting these immune-related proteins to synaptic and sleep regulation.

We next performed pairwise comparisons between 2xBRP control and 1xBRP, 3xBRP, or 4xBRP flies to identify proteins with significant changes in abundance. In the synaptosome P2 fraction, protein abundance showed a general trend toward downregulation: 4.5% in 1xBRP (247/5453), 4.6% in 3xBRP (253/5453), and 6.0% in 4xBRP (329/5453), whereas upregulated proteins comprised a smaller fraction (1.3%, 1.9%, and 4.9%, respectively; Fig. 1*I*). To assess whether proteins previously implicated in sleep regulation were represented among these changes, we performed an enrichment analysis using the keyword “sleep” in functional annotations. Notably, sleep-associated proteins were significantly enriched among the upregulated proteins across all genotypes in the synaptosome P2 fraction (Fig. 1*J*). This included Quiver/Sleepless (Qvr/SSS; Fig. 1*K*), a GPI-anchored Ly-6 family protein that promotes sleep by stabilizing and modulating the Shaker potassium channel (44–47). Qvr/SSS levels were increased in both 3xBRP and 4xBRP flies (Fig. 1*K*), linking BRP directly to a key molecular regulator of sleep. Moreover, several other GPI-anchored Ly-6 family members, including Bero, Bou, CG9338, CG6329, Witty, Crim, and Atilla (48), were also upregulated in 3xBRP and 4xBRP flies (Fig. 1*K*), suggesting broader involvement of this protein family in how BRP-dependent presynaptic plasticity regulates sleep.

### BRP-Dependent Plasticity Triggers Hypophosphorylation of Presynaptic Proteins.

**Fig. 2.**
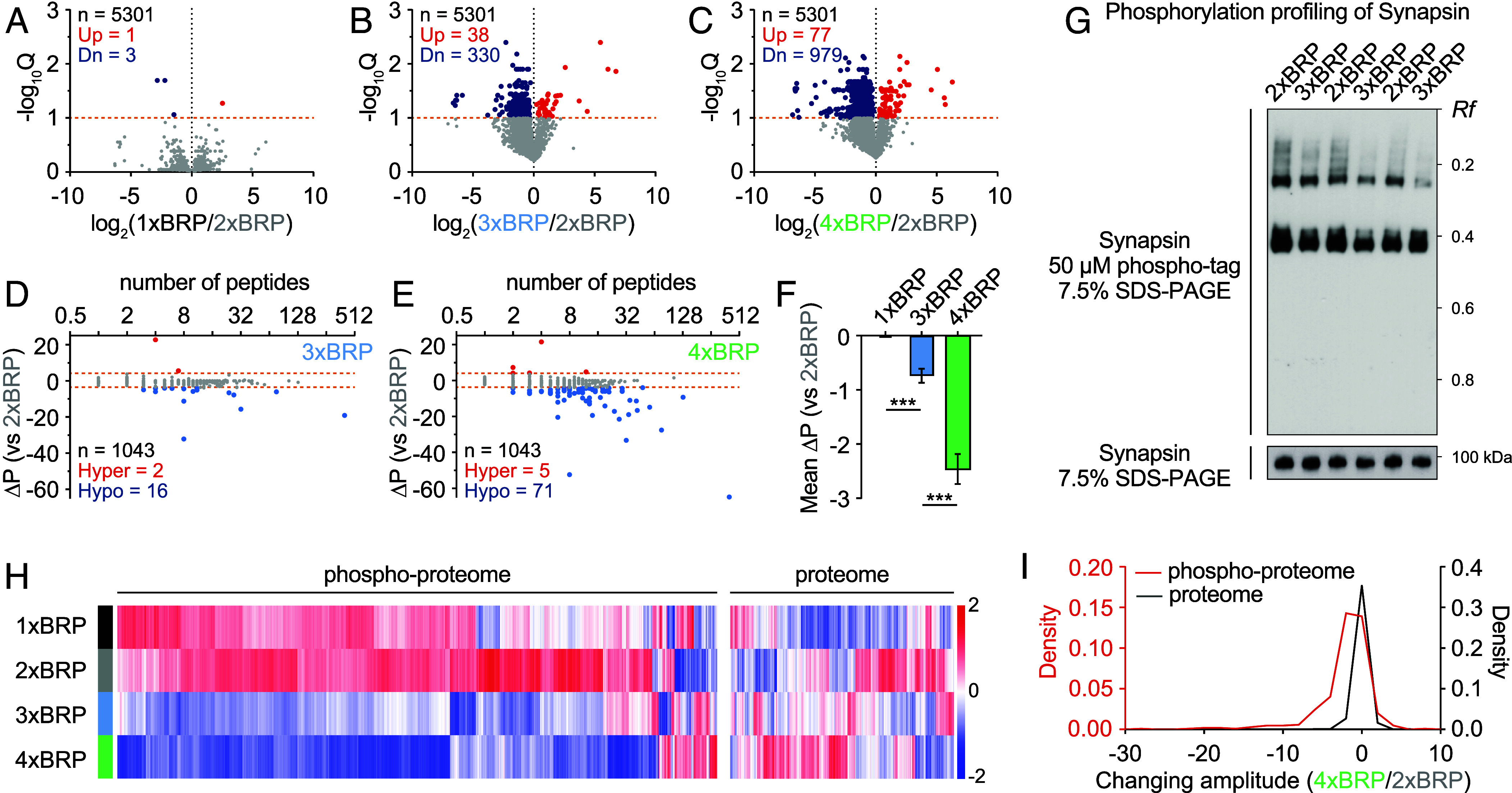
BRP-dependent presynaptic plasticity drives synaptic dephosphorylation. (*A*–*C*) Volcano plots showing changes in phospho-peptides comparing 1xBRP (*A*), 3xBRP (*B*), or 4xBRP (*C*) to 2xBRP control in the synaptosome P2 fraction. Numbers of upregulated and downregulated, and the total phospho-peptides detected are shown. Multiple unpaired *t* tests followed by FDR correction. (*D* and *E*) Protein phosphorylation state analysis of 3xBRP (*D*) and 4xBRP (*E*) compared to 2xBRP control. Numbers of hyperphosphorylated and hypophosphorylated proteins, and the total number of proteins detected are indicated. (*F*) Cumulative phosphorylation state changes of phospho-proteins when comparing 1xBRP, 3xBRP, or 4xBRP to 2xBRP control in the synaptosome P2 fraction. Kruskal–Wallis test followed by Dunn’s multiple comparisons test. (*G*) Phosphorylation of Synapsin in the synaptosome P2 fraction of 3xBRP compared to 2xBRP control was assessed by phospho-tag SDS-PAGE followed by immunoblotting (three independent replicates are shown). In parallel, synaptosome Synapsin protein abundance was assessed by SDS-PAGE and immunoblotting. (*H*) Significantly regulated phospho-peptides and the abundances of the corresponding proteins. The heat maps show the normalized Z-score. (*I*) Density plots comparing the amplitudes of changes in phospho-peptides and in protein abundances in the synaptosome P2 fraction. ***P < 0.001. Error bars: mean ± SEM.

Having analyzed protein abundances across BRP levels in global proteomics, we next turned to phospho-proteomics to examine changes in protein phosphorylation. To account for protein-level effects, phospho-peptide abundances were normalized to their corresponding protein levels measured by the global proteomics, and only proteins detected in both datasets were retained. The final datasets included 4,807 peptides from 1,006 proteins for S1 fraction and 5,301 peptides from 1,043 proteins for P2 fraction (*SI Appendix*, Fig. S1 *C* and *D*). The phospho-proteomic analysis revealed that phosphorylation is drastically downregulated with increasing BRP levels (Fig. 2 *A*–*F* and *SI Appendix*, Fig. S2). In the S1 fraction, when compared to 2xBRP, a major increase of phospho-peptide levels was observed in 1xBRP (*SI Appendix*, Fig. S2*A*), whereas nearly 5% of the detected phospho-peptides were downregulated in 4xBRP flies (*SI Appendix*, Fig. S2*C*). In the synaptosome P2 fraction, downregulation of protein phosphorylation by higher BRP levels was even more pronounced (Fig. 2 *A*–*C*). In 3xBRP flies, we observed that 330 out of 5,301 phospho-peptides showed a significantly decreased phosphorylation when compared to 2xBRP control animals (Fig. 2*B*). Consistently, in 4xBRP animals, an even more dramatic drop in phosphorylation level was detected (Fig. 2*C*): 18% of the detected phospho-peptides were downregulated, while only 1.5% were upregulated.

In the phospho-proteomic quantification, the number of identified phospho-peptides for each protein ranges from 1 to 603, with individual peptides containing one or multiple phosphorylation residues. The cumulative phosphorylation status of a protein is known to influence its function (49). To distinguish whether the observed global hypophosphorylation trend reflects broad changes across many proteins or is only driven by a small number of highly multiphosphorylated proteins, we calculated the cumulative phosphorylation state change (∆P) of each protein by summing the log_2_(fold-change) values of all its significantly regulated phospho-peptides (15). In this analysis, proteins with ∆P > 4 were classified as hyperphosphorylated proteins, while those with ∆P < −4 were classified as hypophosphorylated proteins, based on an average SD of 1.96 across all the samples.

In the S1 fraction, a mild change of protein cumulative phosphorylation was observed (*SI Appendix*, Fig. S2 *D* and *E*). By averaging the cumulative phosphorylation level of each protein in 1xBRP, 3xBRP, and 4xBRP relative to 2xBRP *wt* control, we observed a global shift toward dephosphorylation with increasing BRP levels (*SI Appendix*, Fig. S2*F*). In contrast, the synaptosome P2 fraction showed more remarkable dephosphorylation, with 16 and 71 hypophosphorylated proteins identified in 3xBRP and 4xBRP, respectively (Fig. 2 *D* and *E*). Consistently, with the average ∆P assessment, we demonstrate a larger extent of global dephosphorylation level driven by higher BRP levels in the synaptosome P2 fraction than in the S1 fraction (Fig. 2*F* and *SI Appendix*, Fig. S2*F*).

Phosphorylation changes identified in the phospho-proteomic were further confirmed using phospho-tag SDS-PAGE analysis (15, 50). Synapsin (Syn), an evolutionarily conserved protein whose total protein level did not significantly differ in synaptosome P2 fraction between 2xBRP and 3xBRP, showed a dramatic reduction of heavily phosphorylated species in 3xBRP (Fig. 2*G* and *SI Appendix*, Fig. S3*A*). We also attempted to assess phosphorylation changes under sleep deprivation. However, likely due to the low abundance of phospho-Synapsin, heavily phosphorylated Synapsin species were not detectable in phospho-tag SDS-PAGE from unenriched head homogenates (*SI Appendix*, Fig. S3*C*), different from enriched synaptosome samples (Fig. 2*G* and *SI Appendix*, Fig. S3*B*). Because very large number of flies are needed for integrated proteomics and phospho-proteomics analysis of synaptosomal materials, we were unable to perform similar omics analysis for sleep-deprived flies.

Notably, these hypophosphorylation changes are independent of protein abundance, as the expression levels of the corresponding proteins did not show consistent directional changes (Fig. 2*H*). Moreover, the magnitude of changes in the phospho-proteome exceeded those observed in the global proteome (Fig. 2*I*). Cellular compartment and functional enrichment analysis further revealed that annotations related to presynaptic endocytosis and synaptic membrane were overrepresented among hypophosphorylated proteins, indicating an enrichment of phosphorylation changes at presynaptic membrane compartments (*SI Appendix*, Fig. S3*D*). Together, these data support the synapse as a primary compartment of BRP-associated phosphorylation changes.

### Prediction of Kinase Activity Changes Modulated by BRP.

**Fig. 3.**
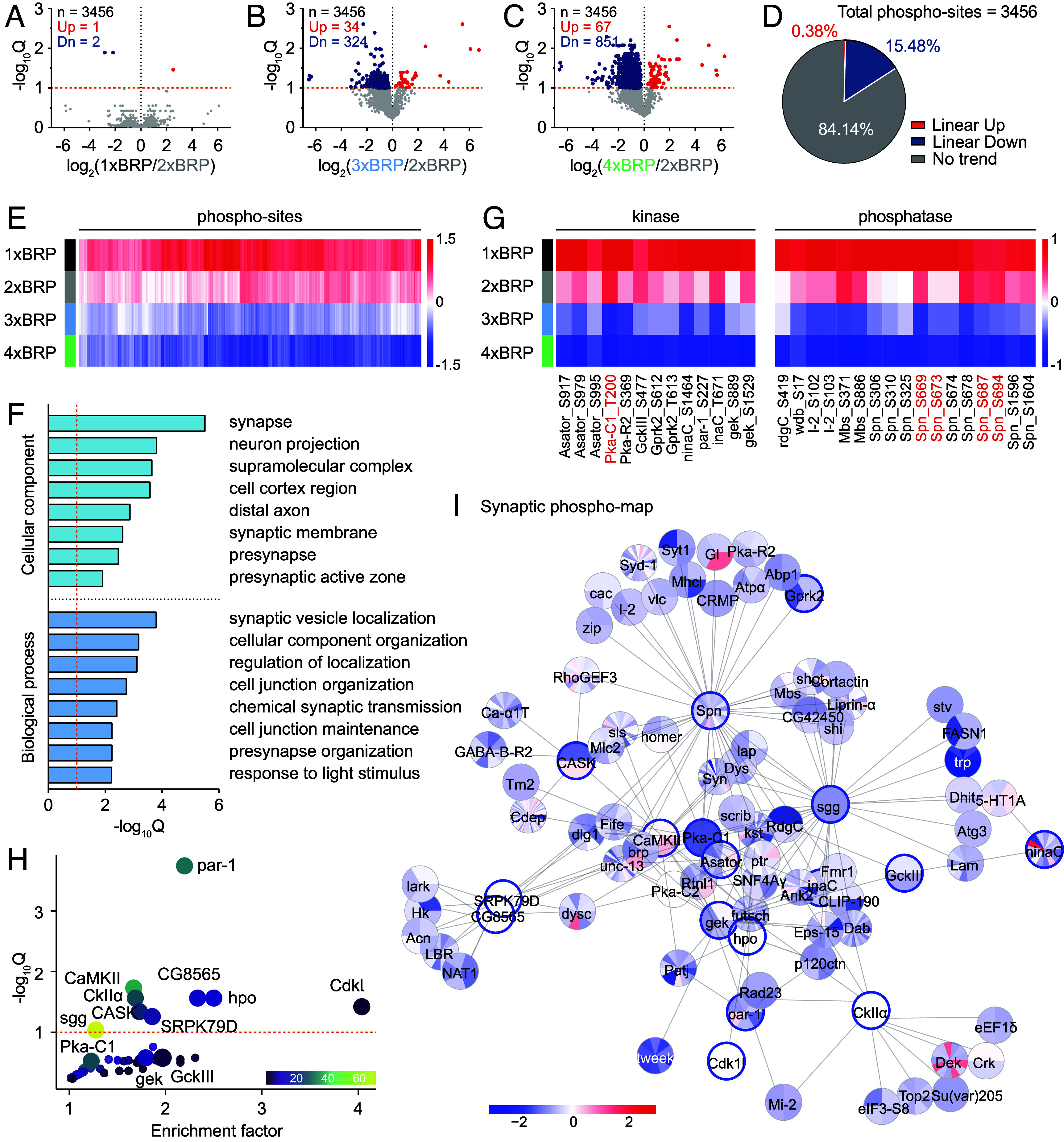
Kinase and phosphatase activity alternations driven by BRP-dependent presynaptic plasticity. (*A*–*C*) Volcano plots showing changes in phospho-sites comparing 1xBRP (*A*), 3xBRP (*B*), or 4xBRP (*C*) to 2xBRP control in the synaptosome P2 fraction. Numbers of upregulated and downregulated, and the total number of identified phospho-sites are shown. Multiple unpaired *t* tests followed by FDR correction. (*D*) The distribution of phospho-sites with linear changes in their phosphorylation levels (positively or negatively) with BRP in the synaptosome P2 fraction. (*E*) Phosphorylation levels of phospho-sites scaled down with BRP. The heat maps show the normalized Z-score. (*F*) Enriched GO terms in the biological process and cellular component categories of proteins containing residues whose phosphorylation levels negatively correlated to BRP levels. (*G*) Phospho-sites on kinases and phosphatases with phosphorylation levels scaled down with BRP. The heat maps show the normalized Z-score. (*H*) Kinases predicted to be involved in synaptic hypophosphorylation of BRP-dependent presynaptic plasticity. The enrichment factor is plotted against significance indicated by the −log10(q-valve). The color gradient of dots represents the number of linear-changing phospho-sites. Hypergeometric tests followed by FDR correction. (*I*) Synaptic phospho-interactome-based predicted kinase–substrate relationships and protein–protein interaction. Wedge colors represent the log_2_(fold-change) in phosphorylation levels at each detected phospho-site of a given protein when comparing 4xBRP to 2xBRP control.

To dissect the role of site-specific phosphorylation modulation and the kinases involved here, we combined normalized phospho-peptide abundances that map to the same modification residue to generate normalized abundances of each phospho-site. In total, 3,456 nonredundant high probability phospho-sites (location probability > 75%) were identified in the synaptosome P2 fraction, predominantly on serine (87.1%) (*SI Appendix*, Fig. S1 *E* and *F*). We again observed lower site-specific phosphorylation levels with increasing BRP levels (Fig. 3 *A*–*C*). We further applied a linear regression test to all the phospho-sites and identified 535 sites (15.48%), whose abundances were significantly and negatively correlated with BRP levels, while only 13 sites scaled positively with BRP levels (Fig. 3 *D* and *E*). Proteins containing these hypophosphorylated sites were highly enriched in presynaptic components and are functionally implicated in regulating active zone organization and synaptic transmission, supported by significantly enriched structural and functional GO terms, including presynapse, distal axon, presynaptic active zone, synaptic vesicle localization, and presynapse organization among the top hits (Fig. 3*F*). Notably, no postsynapse-specific GO terms were detected, underscoring the selective impact of BRP-dependent plasticity on presynaptic phosphorylation dynamics. Importantly, several regulated phospho-sites were located on catalytic or regulatory subunits of kinases and phosphatases (Fig. 3*G*), suggesting potential regulations of their activities.

Of particular relevance to sleep homeostasis, we observed the dephosphorylation of a key conserved site of the catalytic subunit of the wake-promoting Protein Kinase A (PKA) (20, 22), the Thr-200 (corresponding to the mammalian Thr-198) (Fig. 3*G* and *SI Appendix*, Fig. S4*A*), located within the activation loop of the catalytic subunit of PKA. Since the phosphorylation at Thr-200 is essential for PKA enzymatic activity (51), the observed negative correlation between BRP levels and PKA Thr-200 phosphorylation suggests that high BRP dosage suppresses PKA activity. In parallel, we detected 11 residues on a regulatory subunit of Protein Phosphatase 1 (PP1), Spinophilin (Spn), whose phosphorylation levels decreased linearly with BRP levels (Fig. 3*G*). Four of these sites, Ser-669, Ser-673, Ser-687, and Ser-694, are evolutionarily conserved and located within the actin-binding domain of Spn (*SI Appendix*, Fig. S4*B*). Phosphorylation within this region potentially affects the subcellular localization of Spn (52). This is particularly relevant in this context, as Spn-dependent control of presynaptic actin organization has been shown to modulate the structural and functional plasticity of active zones in *Drosophila* (53), further linking BRP-driven plasticity to the modulation of presynaptic architecture and function.

### Generating a Presynaptic Plasticity-Mediated Synaptic Phospho-Map.

To trace the kinase signaling pathways responsible for the linear changes in phosphorylation levels at the detected phospho-sites triggered by BRP-driven presynaptic plasticity, we sought to identify the upstream kinases. However, the limited coverage of curated phospho-site annotations for *Drosophila* within currently available databases constrains direct identification of kinase–substrate relationships. To overcome this, we employed the Group-based Prediction System (GPS) v5.0 (54) which allowed us to predict kinase–substrate relations for all phospho-sites identified in our phospho-proteomic dataset. Using a stringent threshold (false discovery rate = 2%) to ensure high prediction precision, we generated 53,526 kinase–substrate relationships involving 165 kinases. To further refine these predictions, we integrated protein–protein interaction information from the STRING database (https://version-11-5.string-db.org/), resulting in a high-confidence set of 9,594 kinase–substrate relationships. We then performed an enrichment analysis to identify kinases whose predicted substrates were significantly overrepresented among the phospho-sites linearly downregulated by BRP. This analysis yielded 9 enriched kinases (Fig. 3*H*), including Par-1, which itself harbors a phospho-site whose phosphorylation was negatively correlated with BRP levels (Fig. 3*G*). These kinases represent strong candidates for mediating the presynaptic plasticity-induced synaptic phospho-regulation (Fig. 3*H*). Finally, we integrated these findings into a synaptic phospho-map, depicting the kinase–substrate network of the candidate kinases alongside the Spn interactome (Fig. 3*I*), providing an overview of BRP-induced phosphorylation signaling architecture. This phospho-map identified PKA and Shaggy, along with the PP1 regulatory subunit Spn, as dominant regulators of synaptic phosphorylation, reflected by their extensive predicted synaptic substrates, including multiple kinases.

### Attenuated PKA Kinase Activity Contributes to BRP-Dependent Sleep Phenotypes.

**Fig. 4.**
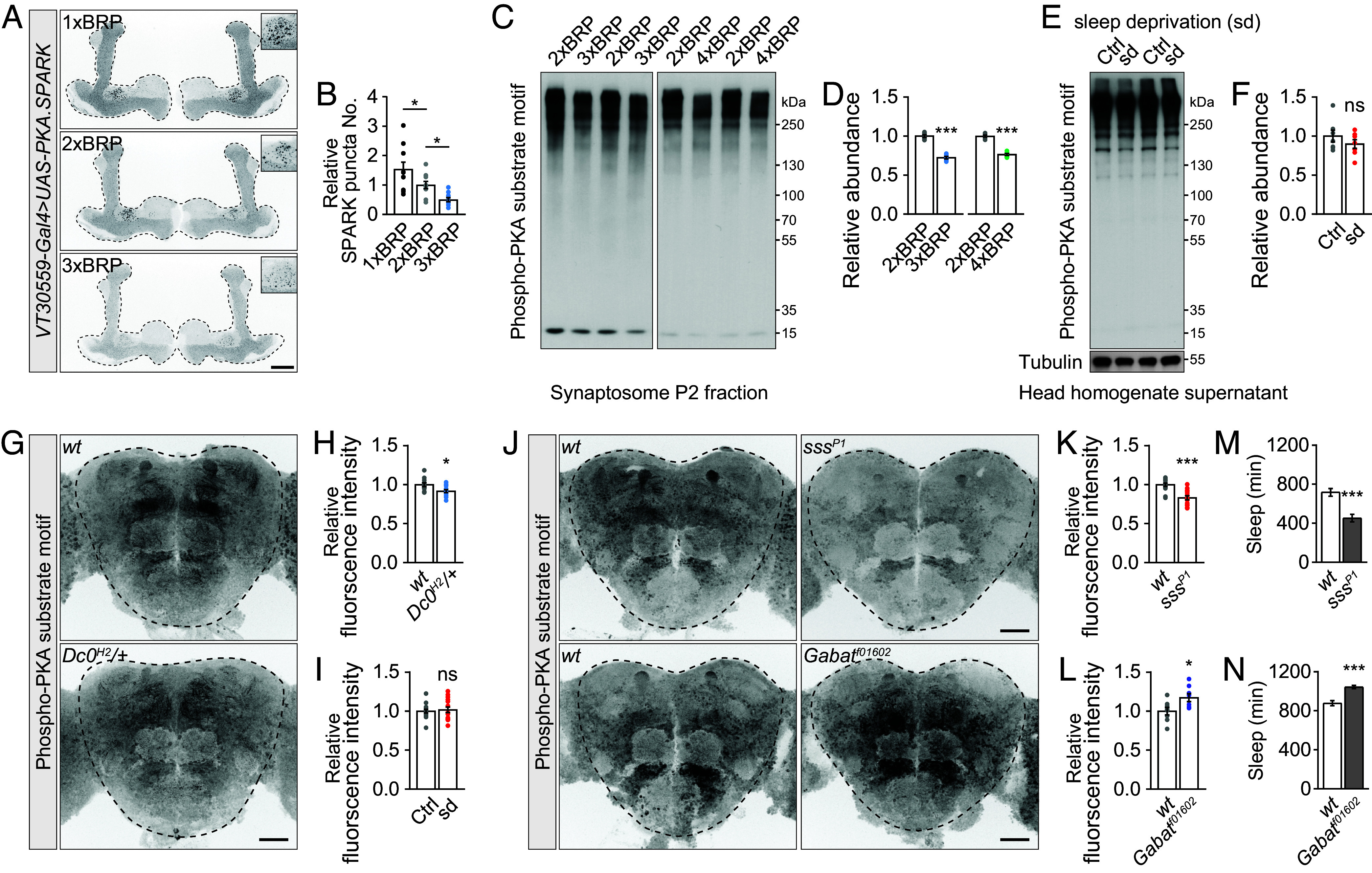
BRP-dependent presynaptic plasticity constrains PKA kinase activity and suppresses the phosphorylation of PKA substrates at the level of synapse. (*A* and *B*) Whole-mount brain immunostaining against GFP for 1 to 3xBRP flies expressing PKA kinase activity sensor PKA.SPARK in the mushroom body driven by *VT30559-Gal4*, including representative images (*A*) and SPARK puncta number (*B*). (Scale bar, 20 μm.) One-way ANOVA with Tukey’s post hoc tests. n = 10. (*C* and *D*) Representative immunoblots (*C*) and statistics (*D*) of synaptosomal samples of 3xBRP or 4xBRP compared to 2xBRP control with antibody specific for PKA target phosphorylation motifs. Student *t* tests. n = 7. (*E* and *F*) Representative immunoblots (*E*) and statistics (*F*) of head homogenate supernatant of sleep-deprived (sd) *wt* flies compared to rested *wt* control (Ctrl) with antibody specific for PKA target phosphorylation motifs. Student *t* tests. n = 7. (*G* and *H*) Representative images (*G*) and statistics (*H*) of whole-mount brain immunostaining against PKA target phosphorylation motifs for *wt* and *Dc0^H2^*/+ flies. (Scale bar, 50 μm.) Student *t* tests. n = 12. (*I*) Statistics of whole-mount brain immunostaining against PKA target phosphorylation motifs for sleep-deprived (sd) flies compared to rested control (Ctrl) flies. Student *t* tests. n = 15 to 16. (*J*, *K*, and *L*) Representative images (*J*) and statistics of whole-mount brain immunostaining against PKA target phosphorylation motifs for *wt* control, short-sleeping *sss^P1 (^*K*^)^*, and long-sleeping *Gabat^f01602^* (*L*) mutants. (Scale bar, 50 μm.) Student *t* tests. n = 8 to 14. (*M* and *N*) Daily sleep time of *sss^P1^* (*M*) and *Gabat^f01602^* (*N*) mutants is also shown. Student *t* tests. n = 23 to 24. *P < 0.05; **P < 0.01; ***P < 0.001; ns, not significant. Error bars: mean ± SEM.

Among these kinase candidates, we focused on PKA because it harbors a conserved activation-loop phospho-site whose phosphorylation scaled negatively with BRP levels (Fig. 3*G*) and is essential for kinase activity (51). As PKA signaling is prominent in the mushroom body of the fly brain (55), we expressed the PKA activity reporter PKA-SPARK (56) in mushroom body to assess brain PKA activity levels in 1 to 3xBRP flies (Fig. 4*A*). PKA-SPARK forms discrete puncta upon PKA activation from an otherwise diffuse signal (56, 57). Indeed, we observed a linear reduction in SPARK puncta numbers with increasing BRP levels (Fig. 4 *A* and *B*), indicating that elevated BRP levels attenuate PKA kinase activity. Consistent with this, western blotting using a phospho-PKA substrate antibody revealed significantly reduced phospho-PKA substrate level in the synaptosome P2 fraction from 3xBRP and 4xBRP flies compared with 2xBRP controls (Fig. 4 *C* and *D*). Global proteomics did not detect linear changes in the abundance of PKA subunits or other detected kinases across BRP levels (*SI Appendix*, Fig. S5), consistent with the idea that BRP-driven presynaptic plasticity modulates kinase activity rather than kinase abundance.

We next asked whether the reduction in PKA activity observed under high-BRP conditions was more generally related to sleep regulation, and specifically whether it reflected changes in sleep amount or sleep pressure. To address this, we examined overall phospho-PKA substrate levels in head homogenates from sleep-deprived flies. Sleep deprivation resulted in a trend toward reduced phospho-PKA substrate level in head homogenates, although this effect did not reach statistical significance (Fig. 4 *E* and *F*). We further performed anti-phospho-PKA substrate brain immunostaining and validated the staining specificity using heterozygous mutants for *Dc0* (*Dc0^H2^*/+), which encodes the catalytic subunit of PKA. *Dc0^H2^/+* animals displayed a reduction in phospho-PKA substrate level (Fig. 4 *G* and *H*). However, brain staining from sleep-deprived flies did not reveal detectable changes (Fig. 4*I*). This is likely because a single night of sleep loss was insufficient to induce brain-wide changes in PKA activity and/or synapse-specific PKA substrate signals may be masked by strong somatic, nonsynaptic signals in both immunostaining and western blot, consistent with the absence of detectable fluorescence difference between 2xBRP and 3xBRP brains (*SI Appendix*, Fig. S6 *A* and *B*).

We therefore examined whether genetic models of chronically altered sleep states are associated with sustained changes in phospho-PKA substrate levels. In the short-sleeping mutant *sleepless* (*sss*) (45), phospho-PKA substrate staining levels were significantly reduced, whereas in the long-sleeping GABA transaminase (*Gabat*) mutant (58), phospho-PKA substrate signals were increased (Fig. 4 *J*–*N*). Taken together, and considering the wake-promoting role of PKA, these results suggest that hypophosphorylation of PKA substrates observed in animals with higher BRP levels is more closely associated with elevated sleep pressure rather than with increased sleep amount.

**Fig. 5.**
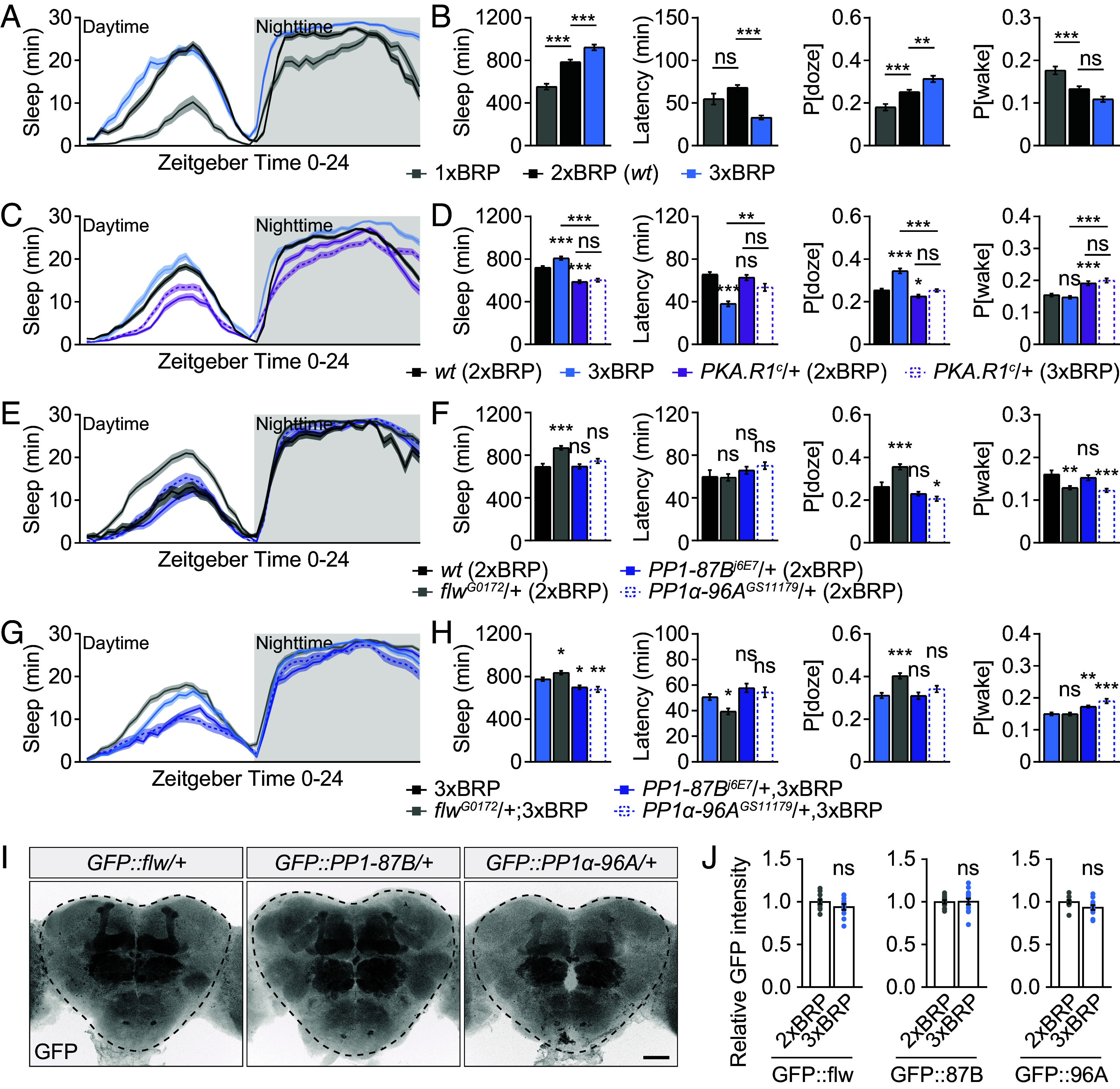
BRP-dependent presynaptic plasticity controls sleep by mediating the activity of PKA kinase and PP1 phosphatases. (*A* and *B*) Sleep profile of 1xBRP, 2xBRP, and 3xBRP flies averaged from measurements over 2 to 4 d, including sleep curves plotted in 30-min bins (*A*), daily sleep amount, sleep latency at ZT12, P[doze] and P[wake] (*B*). n = 40 to 56. (*C* and *D*) Sleep profile of 2xBRP *wt* control and 3xBRP flies, and *PKA.R1^c^*/+ in either 2xBRP or 3xBRP background averaged from measurements over 2 to 4 d, including sleep curves plotted in 30-min bins (*C*), daily sleep amount, sleep latency at ZT12, P[doze] and P[wake] (*D*). n = 59 to 82. (*E* and *F*) Sleep profile of 2xBRP *wt* control flies comparing to PP1-related phosphatase heterozygous mutants averaged from measurements over 2 to 4 d, including sleep curves plotted in 30-min bins (*E*), daily sleep amount, sleep latency at ZT12, P[doze] and P[wake] (*F*). One-way ANOVA with Tukey’s post hoc tests. n = 32 to 56. (*G* and *H*) Sleep profile of 3xBRP flies with or without PP1-related phosphatase heterozygous mutations averaged from measurements over 2 to 4 d, including sleep curves plotted in 30-min bins (*G*), daily sleep amount, sleep latency at ZT12, P[doze] and P[wake] (*H*). One-way ANOVA with Tukey’s post hoc tests. n = 43 to 67. (*I* and *J*) Whole-mount brain immunostaining against GFP for GFP-tagged Flw, PP1-87B, and PP1α-96A, including representative images (*I*) and statistical differences between 2xBRP control and 3xBRP (*J*). (Scale bar, 50 μm.) Student *t* tests. n = 11 to 14. *P < 0.05; **P < 0.01; ***P < 0.001; ns, not significant. Error bars: mean ± SEM.

To test whether reduced PKA activity functionally interacts with BRP-dependent sleep phenotypes, we analyzed sleep behavior of 1 to 3xBRP flies in combination with genetic manipulations that increase PKA activity. Using an independent dataset, we replicated our previous findings (8), showing that flies with 1 to 3xBRP exhibited progressive increases in total sleep amount accompanied by a linear increase in sleep pressure, as measured by P(Doze) (Fig. 5 *A* and *B*). We first sought to enhance PKA activity by pan-neuronal overexpression of the PKA catalytic subunit *Dc0* using *elav-Gal4*. However, *Dc0* overexpression resulted in high posteclosion lethality in 3xBRP background during sleep measurements (*SI Appendix*, Fig. S6*C*). Despite this limitation, we analyzed the available data and compared sleep between 2xBRP and 3xBRP under *Dc0* overexpression (*SI Appendix*, Fig. S6 *D* and *E*). These data suggest that the sleep-promoting effect of 3xBRP is attenuated when PKA activity is enhanced. However, due to the high lethality of 3xBRP;*elav>Dc0* flies, these results should be interpreted with caution. As an alternative approach, we reduced the gene dosage of the regulatory subunit type 1 of PKA (PKA.R1) to its heterozygosity, a manipulation expected to increase PKA activity (55). This resulted in a clear reduction in sleep amounts in 2xBRP control background and fully suppressed the sleep-promoting effects of 3xBRP (Fig. 5 *C* and *D*). Together, these findings indicate that reduced PKA-mediated phosphorylation is required for the expression of BRP-associated sleep phenotypes.

### PP1 Phosphatases Contribute to BRP-Dependent Sleep Phenotypes.

Our phospho-proteomic analysis identified extensive phosphorylation of Spn, with multiple sites exhibiting linear decreases in phosphorylation levels driven by BRP (Fig. 3*G*), implying altered Spn-directed PP1 targeting or substrate specificity. Spn is known to localize PP1 to synapses (59), and its interaction with PP1 depends on a conserved RVxF motif that is necessary and sufficient for PP1 binding in mammals (60). Using AlphaFold3 (61), we predicted that Spn interacts extensively with the most abundant *Drosophila* PP1 subtype (*SI Appendix*, Fig. S7*A*), the PP1-87B (62). We show that Spn binds to the PP1 groove in a conserved manner via its RVxF motif (^1212^PKVRF^1216^) (*SI Appendix*, Fig. S7*A*). To potentially test this interaction, we manipulated PP1 phosphatase levels by generating heterozygous mutants of both *PP1-87B* and *PP1α-96A* and examined for the levels of Spn. We show that Spn level was upregulated when either *PP1-*87B and *PP1α-*96A activity was reduced (*SI Appendix*, Fig. S7 *B* and *C*). An upregulation of an adaptor protein when its phosphatase activity is reduced suggests compensatory mechanism of homeostatic phosphatase activity regulation in PP1.

We next examined whether PP1 catalytic subunits contribute to the elevated sleep phenotypes observed in flies with increased BRP levels. Due to a lack of reliable reporter of phosphatase activity, we were unable to visualize phosphatase activity directly, as we did for PKA kinase (Fig. 4 *A* and *B*), we undertook a behavioral approach by reducing PP1 phosphatase catalytic subunit abundance in 3xBRP flies. PP1α subtype heterozygosity for either *PP1-87B* or *PP1α-96A* significantly suppressed the elevated sleep of 3xBRP flies (Fig. 5 *G* and *H*), while both heterozygous mutants did not show major sleep phenotypes in 2xBRP background (Fig. 5 *E* and *F*). These data suggest that the sleep-promoting effect of 3xBRP is dependent on PP1α activity. Meanwhile, heterozygosity for the PP1β subtype *flw*, which has a nonoverlapping function with the PP1α subtype (62) and been shown required for muscle attachment (63), increased sleep in 2xBRP control background and could further enhance the sleep of 3xBRP animals (Fig. 5 *E*–*H*). These results indicate subtype-specific roles of PP1 catalytic subunits in shaping sleep phenotypes under elevated BRP conditions and that PP1α, but not PP1β, is required for BRP-dependent sleep promotion, likely through protein dephosphorylation. Notably, altering BRP dosage did not result in linear changes in the abundances of PP1 catalytic or regulatory subunits, nor any other detected phosphatases, as assessed by global proteomics (*SI Appendix*, Fig. S8). Consistently, brain imaging of GFP-tagged PP1-87B, PP1α-96A, and Flw revealed no overt differences in expression levels or spatial patterns between 2xBRP and 3xBRP flies (Fig. 5 *I* and *J*), suggesting regulation at the level of phosphatase targeting rather than phosphatase abundance.

### Enrichment of Dephosphorylated Proteins in the Synaptosome.

Having identified and validated synapse-specific changes in phosphorylation dynamics upon BRP titration by using the widely used and well-established synaptosome preparation approach, we finally addressed the functional relevance of synapse-specific phosphorylation dynamics. For this purpose, we conducted tandem mass tags (TMT)-labeled proteome and phospho-proteome analyses on the head homogenate S1 and the synaptosome P2 fractions from 2xBRP *wt* flies. We aimed to map the subcellular distribution of phospho-peptides and to identify kinases and phosphatases enriched at synapses. Compared to S1 fraction, P2 fraction showed a markedly lower overall phosphorylation level (*SI Appendix*, Fig. S9*A*). Specifically, 27.58% detected phospho-peptides exhibited lower phosphorylation level, whereas only 9.50% showed higher phosphorylation level in P2 fraction than in S1 fraction (*SI Appendix*, Fig. S9*A*). This difference is not attributable to changes in protein expression levels, as phospho-peptide abundances were normalized to total protein levels, but rather likely reflects a preferential distribution of dephosphorylated proteins to the synaptic membrane. Consistently, phospho-peptides downregulated in the synaptosome P2 fraction were significantly enriched for synaptic components, particularly for presynaptic but not postsynaptic proteins (*SI Appendix*, Fig. S9 *B* and *C*). To further assess which kinases are more active in the synaptic compartment here, we leveraged the predicted kinase–substrate relationships generated earlier (Fig. 3) and performed kinase substrate enrichment analysis (KSEA) (64). This analysis found that several kinases appeared more active in P2 fraction relative to S1 fraction (*SI Appendix*, Fig. S9*F*). These kinases include Gprk1, Gprk2, and inaC, which contain phospho-sites negatively correlated with BRP levels (Fig. 3*G*), as well as CaMKII, CASK, and Shaggy whose activity was predicted to be weakened by BRP-dependent plasticity (Fig. 3*H*). These findings are largely in line with their differential protein abundances across fractions (*SI Appendix*, Fig. S9*E*). We also identified several receptor tyrosine phosphatases enriched in the synaptosome P2 fraction (*SI Appendix*, Fig. S9*G*). Taking together, these findings suggest that phosphorylation modifications regulate protein subcellular distribution by preferentially localizing dephosphorylated proteins to the presynaptic compartment, particularly the active zone, where their function might be further fine-tuned by locally enriched kinases and phosphatases.

## Discussion

Sleep homeostasis is accompanied by widespread changes in synaptic function, yet the molecular states that link synaptic remodeling to organismal sleep need remain poorly defined. Understanding how synapse-specific molecular programs scale with sleep pressure is therefore essential for connecting cellular plasticity to behavioral regulation.

### Presynaptic Hypophosphorylation as a Molecular State Associated with Sleep Pressure.

In this study, we combined proteomic and phospho-proteomic approaches to perform an in-depth analysis of molecular changes in *Drosophila* with genetically titrated levels of BRP, the master scaffold protein at the active zone. The phospho-proteome of 1 to 4xBRP flies revealed a negative correlation between BRP-driven sleep and global phosphorylation levels (Fig. 2 and *SI Appendix*, Fig. S2). In particular, the synaptosome P2 fraction showed a linear decrease in phosphorylation across over 500 phospho-sites (Fig. 3), indicating a coordinated shift toward presynaptic hypophosphorylation. We interpret this pattern as reflecting altered kinase–phosphatase balance at the synapse, consistent with a system-level modulation of presynaptic phosphorylation state accompanying elevated sleep pressure. These findings point to a strong presynaptic bias of hypophosphorylation within the BRP titration paradigm.

Sleep/wake-driven phospho-proteome oscillations have been previously reported in mice, especially within synaptosomal fractions (14) and synaptic phosphorylation has been shown to decline with sleep (15). In line with these observations, the phospho-proteome of 1 to 4xBRP flies showed decreased presynaptic phosphorylation in animals with elevated BRP levels and increased sleep pressure, supporting the idea that sleep-associated shifts in synaptic phosphorylation state represent a recurring feature across species.

In our genetically defined, titratable model of increased sleep pressure, phosphorylation changes were strongly biased toward presynaptic compartments, with comparatively limited changes detected at postsynapse (Fig. 3*F* and *SI Appendix*, Fig. S3*D*). In particular, hypophosphorylation was largely confined to the active zone, consistent with a presynaptic mode of molecular remodeling accompanying elevated sleep pressure. Together with prior work showing sleep/wake–dependent synaptic phosphorylation dynamics across species, these findings suggest that modulation of presynaptic phosphorylation state represents a general feature associated with sleep homeostasis.

### Kinase–Phosphatase Balance in Synaptic Phosphorylation During Sleep Homeostasis.

Various lines of evidence demonstrated links between synaptic phosphorylation status and the level of sleep (14–18), giving rise to the phosphorylation hypothesis of sleep, which proposes that sleep/wake–dependent kinase activity is associated with changes in synaptic protein phosphorylation linked to sleep/wake regulation (65). In line with this view, multiple kinases have been implicated in the regulation of sleep homeostasis, including PKA. In our dataset, phosphorylation of the activation loop of PKA at Thr-200, was negatively correlated with BRP levels (Fig. 3*G*). The Thr-200 is evolutionarily conserved (*SI Appendix*, Fig. S4*A*), and phosphorylation at this threonine is known to switch PKA from an inactive to an active state (66). Therefore, PKA dephosphorylation at Thr-200 triggered by BRP-dependent presynaptic plasticity indicates reduced PKA activity at synapses. Consistently, the short-sleeping *sss* mutant showed reduced PKA-mediated phosphorylation (Fig. 4 *J*–*L*) and increasing PKA activity suppressed the sleep-promoting effects of 3xBRP (Fig. 5 *C*–*F*). Given that PKA has been shown to promote wakefulness in both flies and mammals (20, 21), its downregulation induced by increasing BRP levels may well represent a conserved molecular mechanism by which elevated sleep pressure suppresses arousal-promoting signaling pathways. A recent study also identified the PKA pathway as a signaling cascade for balancing sleep and memory in *Drosophila* (55). With its broad presynaptic targets being predicted in our analysis (Fig. 3 *G*–*H*), PKA might play an important role in mediating the effects of BRP-dependent presynaptic plasticity in programming a phosphorylation status to regulate sleep homeostasis.

However, the broad synaptic hypophosphorylation observed with increasing BRP levels is unlikely attributed to only a single kinase. Indeed, our analysis identified more than 10 kinases whose predicted activities scale negatively with BRP levels, including SRPK79D, CKII, Shaggy, and Par-1 (Fig. 3 *G*–*H*). The involvement of multiple kinases is consistent with previous findings of murine synaptosome phospho-proteomes, where dynamic oscillation in synaptic phosphorylation was meant to be achieved by the widespread coordinated regulation of kinase activity across sleep/wake states (14).

Alternatively, and not mutually exclusively, BRP-dependent presynaptic plasticity may involve changes in protein phosphatase function or substrate engagement that contribute to synaptic hypophosphorylation. Phosphatases typically act on a broader spectrum of phospho-sites than individual kinases, making them well-suited to coordinate widespread dephosphorylation. In this context, altered phosphatase-associated signaling could complement the coordinated modulation of kinase activity observed with increasing BRP levels and elevated sleep pressure. Supporting this possibility, we observed that multiple phospho-sites on Spinophilin (Spn), a known regulatory protein that targets Protein Phosphatase 1 (PP1) toward specific substrates, were negatively correlated with BRP levels, providing a potential link between BRP-dependent presynaptic plasticity and altered phosphatase-associated signaling at synapses (Fig. 3*G*). The PP1 catalytic subunit can dephosphorylate a wide range of substrates with little motif preference. Substrate selectivity and spatial specificity are instead conferred by regulatory proteins such as Spn, which target PP1 holoenzymes to defined subcellular locations and substrates (67). The phosphorylation of Spn within its actin-binding domain has been shown to disrupt F-actin binding and can influence the subcellular localization of Spn and associated PP1 holoenzymes (52, 68). In our dataset, four conserved serine residues within the Spn actin-binding domain showed phosphorylation levels that scaled negatively with BRP levels (Fig. 3*G* and *SI Appendix*, Fig. S4*B*), suggesting that BRP-dependent presynaptic plasticity may be associated with altered PP1-related signaling at synapses through changes in Spn phosphorylation. Consistent with an involvement of PP1 in this context, we found that genetically reducing the levels of two PP1α catalytic subunits suppressed the elevated sleep phenotype of 3xBRP flies (Fig. 5 *G* and *H*), indicating that PP1α function is required for the expression of BRP-dependent sleep phenotypes. Importantly, PP1 and Spn have been reported to promote sleep in mice (22), suggesting that phosphatase-associated signaling may contribute to sleep regulation across species. Taken together, we speculate that enhanced synaptic PP1α phosphatase activity via higher synaptic substrate affinity mediated by hypophosphorylated Spn acts alongside coordinated kinase modulation, to shape the presynaptic phosphorylation state associated with elevated sleep pressure (Fig. 3*I*).

### Physiological Significance of Presynaptic Phosphorylation for Neurotransmission.

Beyond its role in sleep regulation, broad presynaptic hypophosphorylation is likely to have significant consequences for synaptic physiology. During evoked synaptic transmission, presynaptic active zone proteins undergo extensive and not uniformly directional changes in phosphorylation state (69), suggesting that global shifts in phosphorylation may differentially influence multiple aspects of neurotransmitter release. More generally, presynaptic phosphorylation is known to tune synaptic efficacy by regulating SV pool organization, vesicle cycling, and active zone function. PKA is among the best-characterized kinases in this context. In both mammals and *Drosophila,* PKA-mediated phosphorylation of Syn reduces its affinity for actin, mobilizes SVs from the reserve pool, and enhances neurotransmitter release during sustained activity (70, 71). In addition, phosphorylation of active zone components such as RIM by PKA is required for the induction of long-term presynaptic potentiation (72). Thus, reduced PKA activity is expected to bias synapses toward impaired plasticity. In addition, the PP1 adaptor Spn has been identified as a key regulator of presynaptic plasticity and active zone organization (53, 73, 74). Spn mutants display selective impairments in long term homeostatic presynaptic plasticity (53), linking phosphorylation-dependent coordination of synaptic structure and function to adaptive behavioral states such as sleep.

Together, our comprehensive phospho-proteomic analysis identifies presynaptic hypophosphorylation as a robust molecular signature associated with presynaptic plasticity under conditions of elevated sleep pressure. These findings highlight the importance of posttranslational modifications as locally encoded signals that accompany synaptic remodeling in response to sleep pressure. Notably, several molecular components implicated in this process, such as PKA, PP1, and Spn, are evolutionarily conserved, suggesting that modulation of presynaptic phosphorylation state may represent a broadly relevant principle in sleep–synapse interactions. Future studies dissecting how specific kinases, phosphatases, and their synaptic substrates contribute to these state-dependent changes will be essential for understanding how sleep shapes synaptic function and plasticity across the lifespan.

### Limitations of the Study.

The major finding of our study is that BRP-dependent presynaptic plasticity is associated with a synapse-specific modulation of phosphorylation, governed by the balance between PKA kinase and PP1 phosphatase activity. While our data support a central role for this kinase–phosphatase axis, the precise mechanisms by which broadly distributed enzymes such as PKA and PP1 achieve spatially restricted control of presynaptic phosphorylation remain unclear. In particular, direct presynapse-specific manipulation or tethering of these enzymes is currently not technically feasible, limiting mechanistic resolution at the subcellular level.

More generally, synaptic scaling has been described in multiple contexts, including sleep, learning and memory, neural injury, and presynaptic homeostatic potentiation (8, 75, 76). However, whether these different forms of synaptic plasticity converge on shared phosphorylation-based mechanisms remains unknown. Our findings raise the possibility that presynaptic hypophosphorylation represents one molecular signature of sustained synaptic scaling, but this will require further investigation across systems and conditions.

Due to technical constraints, we limited sample collection for proteomic analysis to ZT6, when sleep differences between genotypes are most pronounced. While this strategy allowed robust detection of BRP-dependent effects, circadian timing is known to influence protein abundance and phosphorylation (14, 77, 78). Future studies will be required to systematically address the interaction between circadian and homeostatic regulation of presynaptic phosphorylation.

Finally, our synaptosomal approach, which enriches for distal synaptic compartments and requires large amounts of materials, does not provide cell-type resolution. While emerging single-cell proteomic strategies may eventually enable such analyses (79), these approaches are not yet well established for the *Drosophila* brain. In the meantime, circuit-level resolution can be addressed using targeted genetic approaches, such as systematic screening with diverse Gal4 driver lines to identify neuronal populations that are particularly sensitive to BRP-dependent modulation.

## Materials and Methods

### *Drosophila* Stocks and Maintenance.

Flies were raised on semidefined medium (Bloomington recipe) under 12/12 h light/dark cycles with 65% humidity at 25 °C. Five- to eight-day-old female flies were used for all experiments except for synaptosome preparation experiments in which mixed populations of both sexes were used. All fly strains were backcrossed to *w^1118^* (iso31, BDSC#5905) background for at least six generations. 1 to 4xBRP flies were generated as previously described (8). Briefly, *wt* (*w^1118^*) control was used as 2xBRP and a *brp* null mutant (*brp^c04298^*, BDSC#85966) was employed to reduce *brp* copy from 2 to 1 (1xBRP). A genomic *brp* P[acman] construct integrated into the 5’ UTR of CG11357 was used to increase *brp* copy number from 2 to 3 or 4 (3xBRP or 4xBRP). Additional fly lines used in this study are as follows: *Dc0^H2^* (BDSC#4101), *sss^P1^* (BDSC#16588), *Gabat^f01602^* (BDSC#85192), *PKA.R1^c^* (BDSC#17792), *elav-Gal4* (BDSC#458), *UAS-Dc0* (BDSC#35554), *flw^G0172^* (BDSC#12287), *PP1-87B^j6E7^* (BDSC#12129), *PP1α-96A^GS11179^* (BDSC#23697), *GFP::flw*, *GFP::PP1-87B, and GFP::PP1α-96A* (80).

### Subcellular Fractionation, LC–MS/MS, and Integrated Omics Analysis.

All fly samples were collected at ~zeitgeber time 6 (ZT6), snap-frozen immediately in liquid nitrogen and stored at −80 °C. Biochemical subcellular fraction preparation was performed based on a protocol described previously (31). Global and IMAC enriched phospho-peptides corresponding to 0.5 µg P2 or 0.25 µg S1 (based on the AAA) and 1.4 µg P2 or 1.0 µg S1 (based on the Nanodrop), respectively, were analyzed by nano-LC–MS/MS using an Ultimate 3000 nano RSLC system coupled to a Orbitrap Lumos MS. All phospho-peptides enrichment experiments were performed on an AssayMAP liquid handling platform (Agilent) using the Phosphopeptide Enrichment 2.0 protocol with Fe(III)-NTA 5 μL cartridges (G5496-60085). All the raw datasets for either S1 or P2 fraction were analyzed simultaneously with the Proteome Discoverer (PD) software 2.4 using the precursor-based label-free quantitation workflow nodes. More experimental details for synaptosome preparation and omics analyses are included in Supporting Information.

Lists of significantly upregulated and downregulated proteins from proteomic and phospho-proteomic experiments were uploaded to Metascape (https://metascape.org/) and enrichment analysis was performed for Gene Ontology (GO) Biological processes, Cellular components, and KEGG Pathway with a cut-off of *P* < 0.01, enrichment factor > 1.5, and number of hits > 2.

### Kinase Prediction and Protein Phosphorylation State Analysis.

Kinase prediction for all the detected phospho-sites was performed with a Group-based Prediction System (GPS) v5.0 (54). Predictors for 165 kinases with a high threshold (false positive rate = 2%) were applied to all the phospho-sites. Then the predicted kinase–substrate relationships were filtered with protein–protein interaction information from STRING database (https://version-11-5.string-db.org/). The kinase enrichment analysis was performed in two ways, with the hypergeometric test and KSEA-base method (64).

The cumulative phosphorylation state change (∆Ps) analysis for detected proteins was performed as previously described with slight modifications (15). The abundances of phospho-peptides were normalized to the individual protein levels. Only the proteins found in both proteomic and phospho-proteomic experiments were included in this analysis.

### Phospho-tag SDS–PAGE and Immunoblotting.

For synaptosome samples, protein concentration was determined by BCA assay and equal amounts of protein were loaded into either phospho-tag or normal SDS-PAGE gels. For head homogenate supernatant of sleep-deprived samples, the relative amounts of protein were determined by Tubulin as loading control. By binding to the phospho-tag chemical, Phospho-tag SDS-PAGE separates phosphorylated and nonphosphorylated proteins during electrophoresis based on their phosphorylation levels (15, 50). The Rf value of 1.0 is defined as the position of bromophenol blue dye. After electrophoresis, proteins on SDS-PAGE were transferred to nylon membrane for further immunoblotting.

Western blotting was performed as previously described (8). The following primary antibodies were used: rabbit anti-BRP^D2^ (81) (1:100,000), rabbit anti-Hsp60 (Enzo #ADI-SPA-805, 1:3,000), mouse anti-Synapsin (DSHB #3C11, 1:1,000), and rabbit anti-PKA substrate (Cell Signaling #9624, 1:10,000). Secondary horseradish peroxidase (HRP)-conjugated goat anti-rabbit or anti-mouse antibodies (Jackson ImmunoResearch, 1:5,000) were used for detecting the protein signals. The developed films without saturation were scanned by an EPSON V330 scanner and saved in 16-bit grayscale tiff format.

### Whole-Mount Brain Immunostaining, Confocal Microscopy, and Image Analysis.

Whole-mount brain immunostaining was performed as previously described (8). The following primary antibodies were used: rabbit anti-GFP conjugated with Alexa 488 (Cat# A-21311, 1:500); chicken anti-GFP (Abcam #13970, 1:1,500); rabbit anti-PKA substrate (Cell Signaling #9621, 1:1,000); rabbit anti-Spn (73) (1:500). Goat anti-rabbit Alexa 488 and 647, and goat anti-chicken Alexa 488 were diluted at 1:300 for secondary antibody incubation.

Whole-mount adult brain samples were imaged on a Leica TCS SP8 confocal microscope from Leica Microsystems, and images were obtained using the Leica Application Suite X. Image stacks were processed and analyzed with Fiji (https://fiji.sc/). For analyzing PKA SPARK puncta, image stacks of mushroom body lobes were converted to two-dimensional images using max Z-projection. The average gray pixel value of PKA SPARK was measured within a ROI of mushroom body lobes segmented automatically after background subtraction, image filtering, and thresholding processes in ImageJ (Fiji). The SPARK puncta were detected using “Find Maxima” (55). For PKA substrate and Spn staining, analysis was performed similarly, except that the projection of a stack of confocal images of adult brains using “average intensity” was chosen and a region of interest was drawn to determine the mean intensity of all the pixels of this region of interest to analyze the fluorescence intensity.

### Sleep Measurements.

Sleep and sleep deprivation experiments were performed exactly as previously reported (8, 55). Briefly, sleep of single female flies was measured *Drosophila* Activity Monitors (DAM2) from Trikinetics Inc. in 12/12 h light/dark cycle with 65% humidity at 25 °C. Three to four-day-old single female flies were loaded into Trikinetics glass tubes (5 mm inner diameter and 65 mm length) with 5% sucrose and 2% agar in one side of the tube. A period of immobility without locomotor activity counts lasting for at least 5 min was determined as sleep (82). For sleep deprivation, DAM2 monitors were fixed onto a Vortexer Mounting Plate (Trikinetics) on an Analog Multi-Tube Vortexer controlled by a Trikinetics LC4 light controller and acquisition software. Pulses of vortex lasting for 1.2 s were applied to the flies randomly with interpulse intervals between 0 s and 40 s to fully deprive sleep from ZT12 to ZT24 (nighttime). Sleep data were analyzed using the Sleep and Circadian Analysis MATLAB Program (SCAMP) (83).

### Data Representation and Statistics.

Graphs were created with Python, R, PyMOL, GraphPad Prism, and Adobe Illustrator. Statistical analysis was performed using GraphPad Prism 8, Microsoft Excel and R. Statistical methods are stated within figure legends and the statistical details used in the bioinformatic analysis were described in previous sections.

## Supplementary Material

Appendix 01 (PDF)

## Data Availability

The mass spectrometry proteomics data have been deposited to the ProteomeXchange Consortium via the PRIDE (84) partner repository with the dataset identifier PXD067034 and 10.6019/PXD067034. All other data are included in the manuscript and/or *SI Appendix*.

## References

[1] P. J. Shaw, G. Tononi, R. J. Greenspan, D. F. Robinson, Stress response genes protect against lethal effects of sleep deprivation in *Drosophila*. Nature **417**, 287–291 (2002). 12015603 10.1038/417287a

[2] M. A. Miller, The role of sleep and sleep disorders in the development, diagnosis, and management of neurocognitive disorders. Front. Neurol. **6**, 224 (2015). 26557104 10.3389/fneur.2015.00224 PMC4615953

[3] T. C. Sudhof, The presynaptic active zone. Neuron **75**, 11–25 (2012). 22794257 10.1016/j.neuron.2012.06.012 PMC3743085

[4] S. Ly, A. I. Pack, N. Naidoo, The neurobiological basis of sleep: Insights from *Drosophila*. Neurosci. Biobehav. Rev. **87**, 67–86 (2018). 29391183 10.1016/j.neubiorev.2018.01.015 PMC5845852

[5] L. de Vivo , Ultrastructural evidence for synaptic scaling across the wake/sleep cycle. Science **355**, 507–510 (2017). 28154076 10.1126/science.aah5982 PMC5313037

[6] G. F. Gilestro, G. Tononi, C. Cirelli, Widespread changes in synaptic markers as a function of sleep and wakefulness in *Drosophila*. Science **324**, 109–112 (2009). 19342593 10.1126/science.1166673 PMC2715914

[7] G. H. Diering , Homer1a drives homeostatic scaling-down of excitatory synapses during sleep. Science **355**, 511–515 (2017). 28154077 10.1126/science.aai8355 PMC5382711

[8] S. Huang, C. Piao, C. B. Beuschel, T. Götz, S. J. Sigrist, Presynaptic active zone plasticity encodes sleep need in *Drosophila*. Curr. Biol. **30**, 1077–1091.e1075 (2020). 32142702 10.1016/j.cub.2020.01.019

[9] S. Huang, C. Piao, C. B. Beuschel, Z. Zhao, S. J. Sigrist, A brain-wide form of presynaptic active zone plasticity orchestrates resilience to brain aging in *Drosophila*. PLoS. Biol. **20**, e3001730 (2022). 36469518 10.1371/journal.pbio.3001730 PMC9721493

[10] A. Suppermpool, D. G. Lyons, E. Broom, J. Rihel, Sleep pressure modulates single-neuron synapse number in zebrafish. Nature **629**, 639–645 (2024). 38693264 10.1038/s41586-024-07367-3 PMC11096099

[11] G. Tononi, C. Cirelli, Sleep and the price of plasticity: From synaptic and cellular homeostasis to memory consolidation and integration. Neuron **81**, 12–34 (2014). 24411729 10.1016/j.neuron.2013.12.025 PMC3921176

[12] G. Tononi, C. Cirelli, Sleep and synaptic down-selection. Eur. J. Neurosci. **51**, 413–421 (2020). 30614089 10.1111/ejn.14335 PMC6612535

[13] T. Sawada , Prefrontal synaptic regulation of homeostatic sleep pressure revealed through synaptic chemogenetics. Science **385**, 1459–1465 (2024). 39325885 10.1126/science.adl3043

[14] F. Brüning , Sleep-wake cycles drive daily dynamics of synaptic phosphorylation. Science **366**, eaav3617 (2019). 31601740 10.1126/science.aav3617

[15] Z. Wang , Quantitative phosphoproteomic analysis of the molecular substrates of sleep need. Nature **558**, 435–439 (2018). 29899451 10.1038/s41586-018-0218-8 PMC6350790

[16] T. Honda , A single phosphorylation site of SIK3 regulates daily sleep amounts and sleep need in mice. Proc. Natl. Acad. Sci. U.S.A. **115**, 10458–10463 (2018). 30254177 10.1073/pnas.1810823115 PMC6187192

[17] C. Mikhail, A. Vaucher, S. Jimenez, M. Tafti, ERK signaling pathway regulates sleep duration through activity-induced gene expression during wakefulness. Sci. Signal. **10**, eaai9219 (2017). 28119463 10.1126/scisignal.aai9219

[18] H. Funato , Forward-genetics analysis of sleep in randomly mutagenized mice. Nature **539**, 378–383 (2016). 27806374 10.1038/nature20142 PMC6076225

[19] S. J. Kim , Kinase signalling in excitatory neurons regulates sleep quantity and depth. Nature **612**, 512–518 (2022). 36477539 10.1038/s41586-022-05450-1

[20] J. C. Hendricks , A non-circadian role for cAMP signaling and CREB activity in *Drosophila* rest homeostasis. Nat. Neurosci. **4**, 1108–1115 (2001). 11687816 10.1038/nn743

[21] X. Yin , Calcineurin governs baseline and homeostatic regulations of non–rapid eye movement sleep in mice. Proc. Natl. Acad. Sci. U.S.A. **122**, e2418317122 (2025). 39847332 10.1073/pnas.2418317122 PMC11789068

[22] Y. Wang , Postsynaptic competition between calcineurin and PKA regulates mammalian sleep–wake cycles. Nature **636**, 412–421 (2024). 39506111 10.1038/s41586-024-08132-2

[23] J. T. Weiss, J. M. Donlea, Sleep deprivation results in diverse patterns of synaptic scaling across the *Drosophila* mushroom bodies. Curr. Biol. **31**, 3248–3261.e3243 (2021). 34107302 10.1016/j.cub.2021.05.018 PMC8355077

[24] J. T. Weiss, M. Z. Blundell, P. Singh, J. M. Donlea, Sleep deprivation drives brain-wide changes in cholinergic presynapse abundance in *Drosophila melanogaster*. Proc. Natl. Acad. Sci. U.S.A. **121**, e2312664121 (2024). 38498719 10.1073/pnas.2312664121 PMC10990117

[25] S. Liu, Q. Liu, M. Tabuchi, M. N. Wu, Sleep drive is encoded by neural plastic changes in a dedicated circuit. Cell **165**, 1347–1360 (2016). 27212237 10.1016/j.cell.2016.04.013 PMC4892967

[26] M. Joyce , Divergent evolution of sleep in *Drosophila* species. Nat. Commun. **15**, 5091 (2024). 38876988 10.1038/s41467-024-49501-9 PMC11178934

[27] R. J. Kittel , Bruchpilot promotes active zone assembly, Ca^2+^ channel clustering, and vesicle release. Science **312**, 1051–1054 (2006). 16614170 10.1126/science.1126308

[28] S. Huang, S. J. Sigrist, Presynaptic and postsynaptic long-term plasticity in sleep homeostasis. Curr. Opin. Neurobiol. **69**, 1–10 (2021). 33333414 10.1016/j.conb.2020.11.010

[29] Y. H. Cho, G. H. Kim, J. J. Park, Mitochondrial aconitase 1 regulates age-related memory impairment via autophagy/mitophagy-mediated neural plasticity in middle-aged flies. Aging Cell **20**, e13520 (2021). 34799973 10.1111/acel.13520 PMC8672789

[30] T. D. Wiggin , Covert sleep-related biological processes are revealed by probabilistic analysis in Drosophila. Proc. Natl. Acad. Sci. U.S.A. **117**, 10024–10034 (2020). 32303656 10.1073/pnas.1917573117 PMC7211995

[31] H. Depner, J. Lutzkendorf, H. A. Babkir, S. J. Sigrist, M. G. Holt, Differential centrifugation-based biochemical fractionation of the *Drosophila* adult CNS. Nat. Protoc. **9**, 2796–2808 (2014). 25393777 10.1038/nprot.2014.192

[32] Y. Zhang, P. Emery, GW182 controls *Drosophila* circadian behavior and PDF-receptor signaling. Neuron **78**, 152–165 (2013). 23583112 10.1016/j.neuron.2013.01.035 PMC3629553

[33] G. P. McNeil, X. Zhang, G. Genova, F. R. Jackson, A molecular rhythm mediating circadian clock output in *Drosophila*. Neuron **20**, 297–303 (1998). 9491990 10.1016/s0896-6273(00)80457-2

[34] V. Sundram , Cellular requirements for LARK in the *Drosophila* circadian system. J. Biol. Rhythms **27**, 183–195 (2012). 22653887 10.1177/0748730412440667 PMC4135585

[35] C. Chen, M. Xu, Y. Anantaprakorn, M. Rosing, R. Stanewsky, Nocte is required for integrating light and temperature inputs in circadian clock neurons of *Drosophila*. Curr. Biol. **28**, 1595–1605.e1593 (2018). 29754901 10.1016/j.cub.2018.04.001

[36] J. C. Darnell , FMRP stalls ribosomal translocation on mRNAs linked to synaptic function and autism. Cell **146**, 247–261 (2011). 21784246 10.1016/j.cell.2011.06.013 PMC3232425

[37] H. R. Monday, S. C. Kharod, Y. J. Yoon, R. H. Singer, P. E. Castillo, Presynaptic FMRP and local protein synthesis support structural and functional plasticity of glutamatergic axon terminals. Neuron **110**, 2588–2606.e2586 (2022). 35728596 10.1016/j.neuron.2022.05.024 PMC9391299

[38] S. Ekengren, D. Hultmark, A family of Turandot-related genes in the humoral stress response of *Drosophila*. Biochem. Biophys. Res. Commun. **284**, 998–1003 (2001). 11409894 10.1006/bbrc.2001.5067

[39] S. Ekengren , A humoral stress response in *Drosophila*. Curr. Biol. **11**, 714–718 (2001). 11369236 10.1016/s0960-9822(01)00203-2

[40] H. Agaisse, U.-M. Petersen, M. Boutros, B. Mathey-Prevot, N. Perrimon, Signaling role of hemocytes in *Drosophila* JAK/STAT-dependent response to septic injury. Dev. Cell. **5**, 441–450 (2003). 12967563 10.1016/s1534-5807(03)00244-2

[41] M. Lagueux, E. Perrodou, E. A. Levashina, M. Capovilla, J. A. Hoffmann, Constitutive expression of a complement-like protein in Toll and JAK gain-of-function mutants of Drosophila. Proc. Natl. Acad. Sci. U.S.A. **97**, 11427–11432 (2000). 11027343 10.1073/pnas.97.21.11427 PMC17216

[42] C. S. Nicolas , The Jak/STAT pathway is involved in synaptic plasticity. Neuron **73**, 374–390 (2012). 22284190 10.1016/j.neuron.2011.11.024 PMC3268861

[43] W. Luo, A. Sehgal, Regulation of circadian behavioral output via a MicroRNA-JAK/STAT circuit. Cell **148**, 765–779 (2012). 22305007 10.1016/j.cell.2011.12.024 PMC3307393

[44] J. M. Humphreys, B. Duyf, M. L. Joiner, J. P. Phillips, A. J. Hilliker, Genetic analysis of oxygen defense mechanisms in *Drosophila melanogaster* and identification of a novel behavioural mutant with a Shaker phenotype. Genome **39**, 749–757 (1996). 8776866 10.1139/g96-094

[45] K. Koh , Identification of SLEEPLESS, a sleep-promoting factor. Science **321**, 372–376 (2008). 18635795 10.1126/science.1155942 PMC2771549

[46] J. W. Wang, J. M. Humphreys, J. P. Phillips, A. J. Hilliker, C. F. Wu, A novel leg-shaking *Drosophila* mutant defective in a voltage-gated K^+^current and hypersensitive to reactive oxygen species. J. Neurosci. **20**, 5958–5964 (2000). 10934243 10.1523/JNEUROSCI.20-16-05958.2000 PMC6772572

[47] M. Wu, J. E. Robinson, W. J. Joiner, SLEEPLESS is a bifunctional regulator of excitability and cholinergic synaptic transmission. Curr. Biol. **24**, 621–629 (2014). 24613312 10.1016/j.cub.2014.02.026 PMC4059605

[48] A. Hijazi , Boudin is required for septate junction organisation in *Drosophila* and codes for a diffusible protein of the Ly6 superfamily. Development **136**, 2199–2209 (2009). 19502482 10.1242/dev.033845

[49] S. J. Humphrey, D. E. James, M. Mann, Protein phosphorylation: A major switch mechanism for metabolic regulation. Trends Endocrinol. Metab. **26**, 676–687 (2015). 26498855 10.1016/j.tem.2015.09.013

[50] E. Kinoshita, E. Kinoshita-Kikuta, K. Takiyama, T. Koike, Phosphate-binding tag, a new tool to visualize phosphorylated proteins. Mol. Cell. Proteomics. **5**, 749–757 (2006). 16340016 10.1074/mcp.T500024-MCP200

[51] H.-X. Jin , Role of phosphorylated Thr-197 in the catalytic subunit of cAMP-dependent protein kinase. J. Mol. Struct. Theochem. **805**, 9–15 (2007).

[52] S. D. Grossman , Spinophilin is phosphorylated by Ca^2+^/Calmodulin-dependent protein kinase II resulting in regulation of its binding to F-actin. J. Neurochem. **90**, 317–324 (2004). 15228588 10.1111/j.1471-4159.2004.02491.x

[53] N. Ramesh , An antagonism between Spinophilin and Syd-1 operates upstream of memory-promoting presynaptic long-term plasticity. eLife **12**, e86084 (2023). 37767892 10.7554/eLife.86084 PMC10588984

[54] C. Wang , GPS 5.0: An update on the prediction of kinase-specific phosphorylation sites in proteins. Genomics Proteomics Bioinf. **18**, 72–80 (2020). 10.1016/j.gpb.2020.01.001 PMC7393560 32200042

[55] S. Huang , Enhanced memory despite severe sleep loss in *Drosophila insomniac* mutants. PLoS Biol. **23**, e3003076 (2025). 40111981 10.1371/journal.pbio.3003076 PMC12135902

[56] Q. Zhang , Visualizing dynamics of cell signaling in vivo with a phase separation-based kinase reporter. Mol. Cell **69**, 334–346.e334 (2018). 29307513 10.1016/j.molcel.2017.12.008 PMC5788022

[57] J. C. Sears, K. Broadie, Temporally and spatially localized PKA activity within learning and memory circuitry regulated by network feedback. eNeuro **9**, ENEURO.0450-21.2022 (2022). 10.1523/ENEURO.0450-21.2022 PMC8982635 35301221

[58] W. F. Chen , A neuron-glia interaction involving GABA transaminase contributes to sleep loss in sleepless mutants. Mol. Psychiatry **20**, 240–251 (2015). 24637426 10.1038/mp.2014.11 PMC4168011

[59] P. B. Allen, C. C. Ouimet, P. Greengard, Spinophilin, a novel protein phosphatase 1 binding protein localized to dendritic spines. Proc. Natl. Acad. Sci. U.S.A. **94**, 9956–9961 (1997). 9275233 10.1073/pnas.94.18.9956 PMC23308

[60] L. C. Hsieh-Wilson, P. B. Allen, T. Watanabe, A. C. Nairn, P. Greengard, Characterization of the neuronal targeting protein spinophilin and its interactions with protein phosphatase-1. Biochemistry **38**, 4365–4373 (1999). 10194355 10.1021/bi982900m

[61] J. Abramson , Accurate structure prediction of biomolecular interactions with AlphaFold 3. Nature **630**, 493–500 (2024). 38718835 10.1038/s41586-024-07487-w PMC11168924

[62] J. Kirchner, S. Gross, D. Bennett, L. Alphey, Essential, overlapping and redundant roles of the *Drosophila* protein phosphatase 1 alpha and 1 beta genes. Genetics **176**, 273–281 (2007). 17513890 10.1534/genetics.106.069914 PMC1893066

[63] Y. Yang , The PP1 phosphatase flapwing regulates the activity of Merlin and Moesin in *Drosophila*. Dev. Biol. **361**, 412–426 (2012). 22133918 10.1016/j.ydbio.2011.11.007

[64] P. Casado , Kinase-substrate enrichment analysis provides insights into the heterogeneity of signaling pathway activation in leukemia cells. Sci. Signal. **6**, rs6 (2013). 23532336 10.1126/scisignal.2003573

[65] K. L. Ode, H. R. Ueda, Phosphorylation hypothesis of sleep. Front. Psychol. **11**, 575328 (2020). 33123055 10.3389/fpsyg.2020.575328 PMC7566165

[66] J. M. Steichen , Global consequences of activation loop phosphorylation on Protein Kinase A. J. Biol. Chem. **285**, 3825–3832 (2010). 19965870 10.1074/jbc.M109.061820 PMC2823524

[67] M. J. Ragusa , Spinophilin directs protein phosphatase 1 specificity by blocking substrate binding sites. Nat. Struct. Mol. Biol. **17**, 459–464 (2010). 20305656 10.1038/nsmb.1786 PMC2924587

[68] L. C. Hsieh-Wilson , Phosphorylation of spinophilin modulates its interaction with actin filaments. J. Biol. Chem. **278**, 1186–1194 (2003). 12417592 10.1074/jbc.M205754200

[69] M. Kohansal-Nodehi, J. J. Chua, H. Urlaub, R. Jahn, D. Czernik, Analysis of protein phosphorylation in nerve terminal reveals extensive changes in active zone proteins upon exocytosis. eLife **5**, e14530 (2016). 27115346 10.7554/eLife.14530 PMC4894758

[70] A. Menegon , Protein kinase A-mediated synapsin I phosphorylation is a central modulator of Ca^2+^-dependent synaptic activity. J. Neurosci. **26**, 11670–11681 (2006). 17093089 10.1523/JNEUROSCI.3321-06.2006 PMC6674776

[71] T. Nuwal, S. Heo, G. Lubec, E. Buchner, Mass spectrometric analysis of synapsins in *Drosophila melanogaster* and identification of novel phosphorylation sites. J. Proteome Res. **10**, 541–550 (2011). 21028912 10.1021/pr100746s

[72] G. Lonart , Phosphorylation of RIM1alpha by PKA triggers presynaptic long-term potentiation at cerebellar parallel fiber synapses. Cell **115**, 49–60 (2003). 14532002 10.1016/s0092-8674(03)00727-x

[73] K. Muhammad , Presynaptic spinophilin tunes neurexin signalling to control active zone architecture and function. Nat. Commun. **6**, 8362 (2015). 26471740 10.1038/ncomms9362 PMC4633989

[74] N. Ramesh , Antagonistic interactions between two neuroligins coordinate pre- and postsynaptic assembly. Curr. Biol. **31**, 1711–1725.e1715 (2021). 33651992 10.1016/j.cub.2021.01.093 PMC8085026

[75] O. Turrel, N. Ramesh, M. J. F. Escher, A. Pooryasin, S. J. Sigrist, Transient active zone remodeling in the Drosophila mushroom body supports memory. Curr. Biol. **32**, 4900–4913.e4904 (2022). 36327980 10.1016/j.cub.2022.10.017

[76] P. Singh, J. M. Donlea, Bidirectional regulation of sleep and synapse pruning after neural injury. Curr. Biol. **30**, 1063–1076.e3 (2020). 32142703 10.1016/j.cub.2019.12.065 PMC7199647

[77] S. B. Noya , The forebrain synaptic transcriptome is organized by clocks but its proteome is driven by sleep. Science **366**, eaav2642 (2019). 31601739 10.1126/science.aav2642

[78] C. Wang , Integrated omics in *Drosophila* uncover a circadian kinome. Nat. Commun. **11**, 2710 (2020). 32483184 10.1038/s41467-020-16514-z PMC7264355

[79] T. Wu , Single-cell proteomic landscape of the developing human brain. Nat. Biotechnol., 10.1038/s41587-025-02980-7 (2026). 41593231

[80] M. T. Bertran , ASPP proteins discriminate between PP1 catalytic subunits through their SH3 domain and the PP1 C-tail. Nat. Commun. **10**, 771 (2019). 30770806 10.1038/s41467-019-08686-0 PMC6377682

[81] W. Fouquet , Maturation of active zone assembly by *Drosophila* Bruchpilot. J. Cell Biol. **186**, 129–145 (2009). 19596851 10.1083/jcb.200812150 PMC2712991

[82] P. J. Shaw, C. Cirelli, R. J. Greenspan, G. Tononi, Correlates of sleep and waking in *Drosophila melanogaster*. Science **287**, 1834–1837 (2000). 10710313 10.1126/science.287.5459.1834

[83] C. G. Vecsey, C. Koochagian, M. T. Porter, G. Roman, D. Sitaraman, Analysis of sleep and circadian rhythms from *Drosophila* activity-monitoring data using SCAMP. Cold Spring Harb. Protoc. **2024**, pdb.prot108182 (2024). 38336392 10.1101/pdb.prot108182 PMC11552080

[84] Y. Perez-Riverol , The PRIDE database at 20 years: 2025 update. Nucleic Acids Res. **53**, D543–D553 (2025). 39494541 10.1093/nar/gkae1011 PMC11701690

