# Supplementary material for: Active zone plasticity couples sleep need to presynaptic hypophosphorylation
Source: Proc Natl Acad Sci U S A. 2026 Jun 8;123(24):e2524065123. doi: 10.1073/pnas.2524065123 (PMC13273273; doi:10.1073/pnas.2524065123)
Supplement: Supplementary file 1 — Appendix 01 (PDF) [file pnas.2524065123.sapp.pdf]

Supporting Information for
Active zone plasticity couples sleep need to presynaptic hypophosphorylation

Chengji Piao,[1,2] Ewelina P. Dutkiewicz,[3] Laxmikanth Kollipara,[3] Albert Sickmann,[3,4]
Sheng Huang,[1,2,*] and Stephan J. Sigrist[1,2,*]

[1]Institute for Biology/Genetics, Freie Universität Berlin, Berlin 14195, Germany
[2]NeuroCure Cluster of Excellence, Charité Universitätsmedizin, Berlin 10117, Germany
[3]Leibniz-Institut für Analytische Wissenschaften, Dortmund 44139, Germany
[4]Medizinische Fakultät, Ruhr-Universität Bochum, Bochum 44801, Germany

*Correspondence: Sheng Huang; Stephan J. Sigrist
Email: sheng.huangflz@gmail.com; stephan.sigrist@fu-berlin.de

This PDF file includes:

Supporting text
Figures S1 to S9
References for SI citations

Supporting text for method details

Biochemical subcellular fractionation

Several pools of 1–4xBRP flies were collected at 5 days post-eclosion. All samples were collected at ~ zeitgeber time 6 (ZT6), snap-frozen immediately in liquid nitrogen and stored at -80 ºC. Biochemical subcellular fraction preparation of the snap-frozen fly heads by differential centrifugation was performed based on a protocol described previously with minor changes (1). Whole frozen flies collected in 50 ml falcon tubes were vortexed and fly heads were isolated from bodies and limbs by size with pre-chilled sieves. Then the fly heads were grounded into a fine powder and suspended in homogenization buffer (320 mM Sucrose, 4 mM HEPES with PhosSTOP™ and cOmplete™ Protease Inhibitor Cocktail, EDTA-free tablets). The suspension was homogenized in a glass-Teflon homogenizer at 900 RPM. The homogenate was then transferred into a flanged centrifugation tube and centrifuged at 1000 g for 10 min at 4 ºC. The supernatant collected at this step, namely S1 fraction, containing brain tissue homogenate with small cell fragments, was further spun at 15000 g for 15 min at 4 ºC. After centrifugation, the pellet (synaptosome P2 fraction) was collected and resuspended with homogenization buffer. S1 and P2 fractions were used in the proteomic and phospho-proteomic analyses. Three biological replicates of each fraction from each condition (1xBRP, 2xBRP, 3xBRP and 4xBRP) were subjected to mass spectrometry quantification using a label-free approach.

Removal of debris and buffer components

Briefly, 10% SDS buffer complete (50 mM Tris-Cl, 150 mM NaCl and protease and phosphatase inhibitors) were added to S1 and reconstituted P2 samples to a final concentration of 1% SDS. Next, all samples were vortexed and centrifuged at 18,000 g at room temperature for 5 min to collect debris. To remove buffer components, the supernatants were collected in new LoBind tubes and aliquots corresponding to ~ 200 µL were subjected to ice-cold EtOH protein precipitation protocol. Samples were diluted 10-fold with cold EtOH, stored at -40°C for 1 h and centrifuged at 15,000 g at 4°C for 30 min. Next, the supernatant was discarded, and the pellets were washed briefly with 500 µL of ice-cold acetone and centrifuged as above. Lastly, the supernatant was discarded, and protein pellets were dried under the laminar flow hood and stored at -80°C until further use.

BCA assay, carbamidomethylation, EtOH protein precipitation and in solution digestion

Each sample was lysed with 1% SDS buffer complete and to degrade any nucleic acids, Benzonase endonuclease together with 2 mM $MgCl_2$ were added and incubated at 37°C for 30 min. Prior Benzonase treatment, P2 samples were briefly subjected to Bioruptor sonication (30 s pulse, 4°C). Samples were centrifuged at 18,000 g at room temperature. Clear supernatant was taken to estimate the protein concentration using BCA assay as per the manufacturer's instructions. Next, samples were subjected to carbamidomethylation i.e., reduction of disulfide bonds with 10 mM DTT at 56°C for 30 min followed by alkylation of free thiol groups with 30 mM IAA at room temperature for 30 min in dark. Approximately, 380 µg (S1) or 500 µg (P2) of protein lysates were subjected to ice-cold EtOH protein precipitation as described above. The pellets were resolubilized in trypsin buffer comprising 50 mM NH_4HCO_3 pH 7.8, 5% acetonitrile, 0.6 M GuHCl, 2 mM $CaCl_2$, and trypsin 1:20 w/w ratio of enzyme to substrate and samples were incubated at 37°C overnight. Tryptic peptides were acidified to pH < 3 with 10% TFA and the digestion efficiency was controlled on a Monolithic-HPLC as described previously (2). Next, samples were desalted using C18 SPEC (15 mg sorbent, Bond Elut Agilent) and vacuum manifold system. Eluates were completely dried in a SpeedVac and reconstituted in 0.1% TFA. Lastly, the peptide concentration of each sample was determined by amino acid analysis (AAA) as previously described (3, 4). Based on the AAA results, peptides of each sample were divided in two parts i.e., ~ 90% for phospho-proteome and ~ 10% for global proteome analyses.

Semi-automated IMAC-based phospho-peptides enrichment

All phospho-peptides enrichment experiments were performed on an AssayMAP liquid handling platform (Agilent) using the *Phosphopeptide Enrichment 2.0* protocol with Fe(III)-NTA 5 µL cartridges (G5496-60085). Approximately, 350 µg (S1) or 450 µg (P2) of each sample was dried and reconstituted in 200 µL of 0.1% TFA in 80% ACN and proceeded with the enrichment procedure. The solvents used were: 0.1% TFA in ACN (priming buffer), 0.1% TFA in 80% ACN (loading and washing buffer), and 1% NH_4OH (elution buffer) into 10% FA. After the enrichment, the acidified eluates were dried completely and reconstituted in 50 µL of 0.1% TFA. An aliquot of each sample was quality controlled on a Monolithic-HPLC, and the peptides concentration was estimated using the Nanodrop spectrophotometer (Thermo).

LC-MS/MS analysis of global peptides and phospho-peptides samples

Global and IMAC enriched phospho-peptides corresponding to 0.5 µg P2 or 0.25 µg S1 (based on the AAA) and 1.4 µg P2 or 1.0 µg S1 (based on the Nanodrop), respectively were analyzed by nano-LC-MS/MS using an Ultimate 3000 nano RSLC system coupled to a Orbitrap Lumos MS (both Thermo Scientific). The samples were measured in a randomized order to minimize technical bias. Briefly, peptides were preconcentrated on a 100 µm x 2 cm C18 trapping column for 5 min using 0.1% TFA (v/v) with a flow rate of 20 µL/min followed by separation on a 75 µm x 50 cm C18 main column (both Acclaim Pepmap nanoviper, Thermo Scientific) with a 90 min, 120 min or 180 min LC gradient ranging from 3-35% of B (84% ACN in 0.1% FA) at a flow rate of 250 nL/min. The Orbitrap Lumos was operated in data-dependent acquisition mode and MS survey scans were acquired from m/z 300 to 1500 at a resolution of 120000 using the polysiloxane ion at m/z 445.12002 as lock mass (5). Precursors were isolated with the Quadrupole at 1.2 m/z (proteomics) and 1.0 m/z (phospho-proteomics) window and MS/MS analysis was performed using a top-speed approach with cycle time of 3 s (proteomics) and 2 s (phospho-proteomics) using higher energy collisional dissociation fragmentation with normalized collision energy at 30% (proteomics) and 32% (phospho-proteomics). MS/MS spectra were acquired in the ion trap using Rapid Ion Trap Scan Rate option (proteomics), whereas for phospho-proteomics MS/MS scans were acquired in the Orbitrap at a resolution of 15000. A dynamic exclusion of 30 s was applied with a mass tolerance of 10 ppm. Automatic gain control (AGC) target values were set to custom for MS and MS/MS scans whereas, normalized AGC were set to 50% (MS) and 20% (MS/MS) and maximum injection times were set to 50 ms (MS) and dynamic (MS/MS), respectively.

Label-free quantitative data analysis

All 12 raw files of each dataset belonging either to S1 or P2 fractions were analyzed simultaneously with the Proteome Discoverer (PD) software 2.4 using the precursor-based label-free quantitation workflow nodes. MS/MS spectra were searched against a merged *Drosophila melanogaster Berkeley* database containing 21919 target entries (3358 Swissprot and 18561 TrEMBL) downloaded in November 2016 using Mascot (Matrix Science) and Sequest algorithms. Trypsin with a maximum of two missed cleavages was selected as enzyme. Carbamidomethylation of Cys was set as fixed, oxidation of Met and acetylation of N-terminus were selected as variable modifications. For phospho-proteome data, phosphorylation on Ser, Thr and, Tyr were set as variable modifications. MS tolerance was set to 10 ppm and MS/MS was 0.5 Da (proteomics) and 0.02 Da (phospho-proteomics), respectively. False discovery rate (FDR) validation on the peptide-spectrum match (PSM) level was done using Percolator node. Peak and feature detection were done by the "Minora" feature detector node using default parameters. For phospho-proteomics data, additionally IMP-ptmRS node was used to calculate the modification site probabilities set to ≥ 75%. In the Consensus workflow of PD, the peptide and protein filters were set to an FDR of 1% and default settings of "Feature Mapper" node were employed. For the "Precursor Ions Quantifier" node, only *unique* peptides were set to use and "Precursor Abundance Based On" was set to *intensity*. "Normalization Mode" was set to *total peptide amount* and for "Scaling" the normalized abundances; *on all average* was selected. "Protein Abundance Calculation" was done by using the *summed abundances* and "Protein Ratio Calculation" was set to *protein abundance based*. For missing values, the "Imputation Mode" was set to *None*. Only those proteins that were quantified with ≥ 1 unique peptide, high confidence settings i.e., 1% FDR on protein, peptide and PSM level and having

a normalized abundance value of ≥ 1.00 in every sample (n=12) were considered for further evaluation. For phospho-proteomics data, only the unique peptide groups that contained phosphorylation modification quantified with high confidence having abundance value of ≥ 1.00 in every sample (n=12) were considered exported from PD software.

Samples preparation for TMT-labeled global proteome and phospho-proteome analyses

S1 and P2 fractions from 2xBRP *wt* in homogenization buffer were first subjected ice-cold EtOH precipitation and acetone wash protocol. P2 fraction was treated with 2% SDS end concentration prior to protein precipitation step. Next, 200 µL of 1% SDS buffer was added to lyse the pellets followed by BCA assay to determine protein concentration. Approximately, 180 µg of protein lysate from each sample was subjected to ice-cold EtOH protein precipitation protocol followed by in solution trypsin digestion. Thus, generated tryptic peptides were quality controlled and desalted as mentioned above.

TMT10plex labelling and TiO2 beads based-enrichment (phospho-proteome) and high pH (8.0) C18 reversed-phase fractionation (global proteome)

Each fraction was labeled with TMT10plex isobaric reagents according to the manufacturer's instructions (Thermo Scientific, Germany) and as previously described (6). After labeling, checking label efficiency and normalizing the amounts; the samples were pooled (multiplexed) and split into two parts i.e., 95% of the sample for the enrichment of phospho-peptides using TiO2-beads based chromatography (7) and 5% for global proteome (8).

LC-MS and data analyses of TMT-labelled peptides

In total 12 fractions from high pH HPLC fractionation (global proteome) and one sample from TiO2 beads chromatography (phospho-proteome) were analyzed on a nano-LC-MS/MS using an Ultimate 3000 nano RSLC system coupled to a Q Exactive HF mass spectrometer (Thermo Scientific) as previously described (6). All MS raw data were processed with Proteome Discoverer (PD) 1.4 (Thermo Scientific) and searched in target/decoy approach against *Drosophila melanogaster Berkeley* database containing 21919 target entries (3358 Swissprot and 18561 TrEMBL) downloaded in November 2016 using Mascot (Matrix Science) and Sequest algorithms using the same set of parameters i.e. precursor and fragment ion tolerances of 10 ppm and 0.02 Da for MS and MS/MS, respectively; trypsin as protease with 2 maximum missed cleavages; carbamidomethylation of Cys (+57.021 Da), TMT10plex on N-terminus and Lys (+229.163 Da) as fixed modifications; oxidation of Met (+15.995 Da), phosphorylation (only for the phospho-proteome data) of Ser, Thr and Tyr as variable modifications. For the phospho-proteome data analysis, the phosphoRS (9) (version 3.1) node was used to score localization probabilities for the identified phospho-sites. Next, the data from PD were exported with the following filter criteria: a false discovery rate (FDR) ≤1% on the PSM level (high confidence setting), search engine rank 1 and only proteins that were quantified with ≥2 unique peptides. For the phospho-proteome data, only unique PSMs filtered with Percolator (10) (FDR <1%) and with a phosphoRS probability ≥90% were considered. Further data analysis was performed as previously described (11).

Differential expression analysis

Different isoforms of the same protein were considered as different proteins for data analysis. Comparisons were made between experimental groups (1xBRP, 3xBRP, and 4xBRP) and *wt* (2xBRP). The multiple unpaired *t-tests* were performed for each comparison (1xBRP/2xBRP, 3xBRP/2xBRP, and 4xBRP/2xBRP) in each fraction (S1 and P2). Proteins with $p < 0.05$ and $\log_2$(fold change) > ± 0.5 were referred to as differentially expressed proteins.

Kinase prediction and enrichment analysis

Kinase prediction for all the detected phospho-sites was performed with a Group-based Prediction System (GPS) v5.0 (12). Predictors for 165 kinases with a high threshold (false positive rate = 2%)

were applied to all the sites. Then the predicted kinase-substrate relationships were filtered with protein-protein interaction information from STRING database (https://version-11-5.string-db.org/). Then kinase enrichment analysis was performed in two ways, with hypergeometric test and KSEA-base method (13).

For all the predicted kinases phosphorylating sites with phosphorylation levels negatively correlated with BRP, the enrichment score of each kinase (*E*), was defined as:

$$E = \frac{m}{M} \div \frac{n}{N}$$

Here, *m* = number of sites scaled down with BRP levels predicted to be phosphorylated by this kinase; *M* = total number of phospho-sites with phosphorylation levels negatively correlated with BRP; *n* = number of all the sites in the fraction predicted to be phosphorylated by this kinase; *N* = number of sites detected in the fraction. Hypergeometric tests were then performed for kinases with an enrichment score greater than 1 using phyper function and *p*-values were corrected using the Benjamini-Hochberg method in R.

In the KSEA-base method, the filtered prediction outputs from the GPS predictor were used as the kinase-substrate annotations database instead of PhosphoSitePlus to increase the coverage of phospho-sites in *Drosophila*. The calculation of the normalized scores and *p*-values of all predicted kinases followed by FDR correction was performed in the KSEA app R package (14).

Protein phosphorylation state analysis

The cumulative phosphorylation state change (ΔPs) analysis for detected proteins was performed as previously described with slight modifications (15). The abundances of phospho-peptides were normalized to the individual protein levels. Only the proteins found in both proteomic and phospho-proteomic experiments were included in this analysis. Multiple comparison tests followed by false discovery rate (FDR) correction were applied to the normalized phospho-peptide data to detect changes of phosphorylation levels in 1xBRP, 3xBRP, and 4xBRP compared to 2xBRP *wt* control. The multiple unpaired *t-tests* were performed for each comparison (1xBRP/2xBRP, 3xBRP/2xBRP, and 4xBRP/2xBRP) in S1 and P2 fractions and the *p*-values were adjusted after FDR (*q*-value) estimation (16). The ΔPs value of a single protein for each comparison was calculated as the sum of $\log_2$(fold change) of all the phospho-peptides detected of that protein with $-\log_{10}$(*q*-value) greater than 1. A cut-off of ±4 as hypo (ΔPs<-4)- or hyper (ΔPs>4)-phosphorylated protein was determined by the average standard deviation for ΔPs from all comparisons (±1.96).

AlphaFold3 structural prediction for PP1/Spn interaction

AlphaFold3 (17) was used to predict the structures of PP1-87B and Spinophilin (Spn), as well as their interaction complex. The amino acid sequences of PP1-87B and Spn were submitted to the AlphaFold server, and available information on phospho-sites was included where applicable. The top-ranked predicted complex model was selected for further analysis. Structural visualization and figure preparation were performed using PyMOL (Schrödinger, LLC). Polar contacts were identified using a distance cutoff of 3.4 Å.

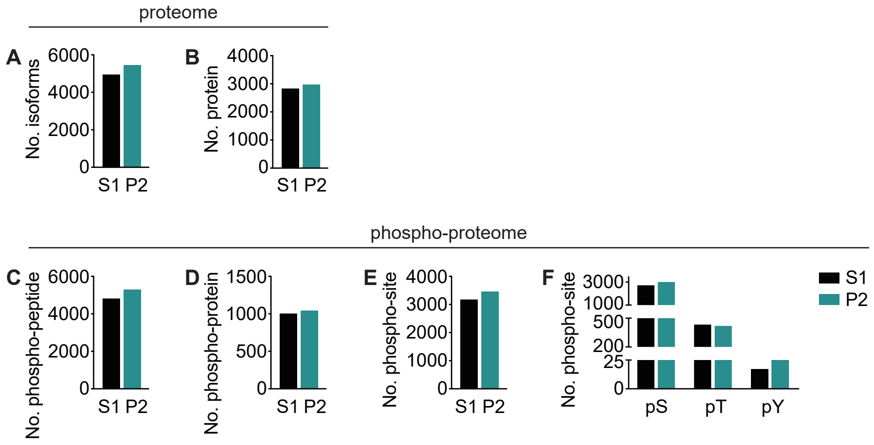

Fig. S1. Number of protein isoforms, proteins, phospho-peptides and phospho-sites detected in S1 and P2 fractions.

(**A** and **B**) Number of detected protein isoforms (**A**) and proteins (**B**).

(**C**–**F**) Number of identified phospho-peptides (**C**), phospho-proteins (**D**), phospho-sites (**E**) and distribution of phosphorylated amino acid residues (pS, phospho-serine; pT, phospho-threonine; pY, phospho-tyrosine) (**F**).

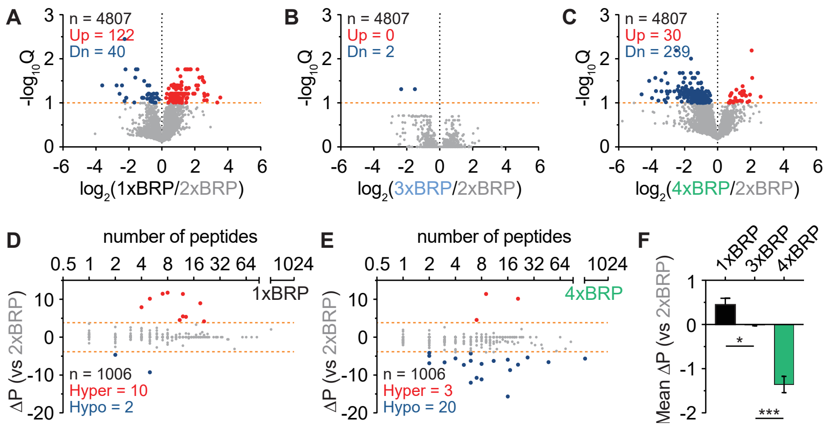

Fig. S2. Protein phosphorylation state in the head homogenate supernatant S1 fraction.

(**A**–**C**) Volcano plots showing changes in phospho-peptides when comparing 1xBRP (**A**), 3xBRP (**B**), or 4xBRP (**C**) to 2xBRP *wt* control in the S1 fraction. Numbers of upregulated and downregulated phospho-peptides, and total phospho-peptides detected are shown. Multiple unpaired t tests followed by FDR correction.

(**D** and **E**) Global protein phosphorylation state analysis of 1xBRP (**D**) and 4xBRP (**E**) compared to 2xBRP *wt* control. Numbers of hyperphosphorylated and hypophosphorylated proteins, and the total number of detected proteins are shown.

(**F**) Cumulative phosphorylation state changes of phospho-proteins when comparing 1xBRP, 3xBRP, or 4xBRP to 2xBRP *wt* control in the head homogenate supernatant S1 fraction. Kruskal-Wallis test followed by Dunn's multiple comparisons test.

*$p < 0.05$; ***$p < 0.001$. Error bars: mean ± SEM.

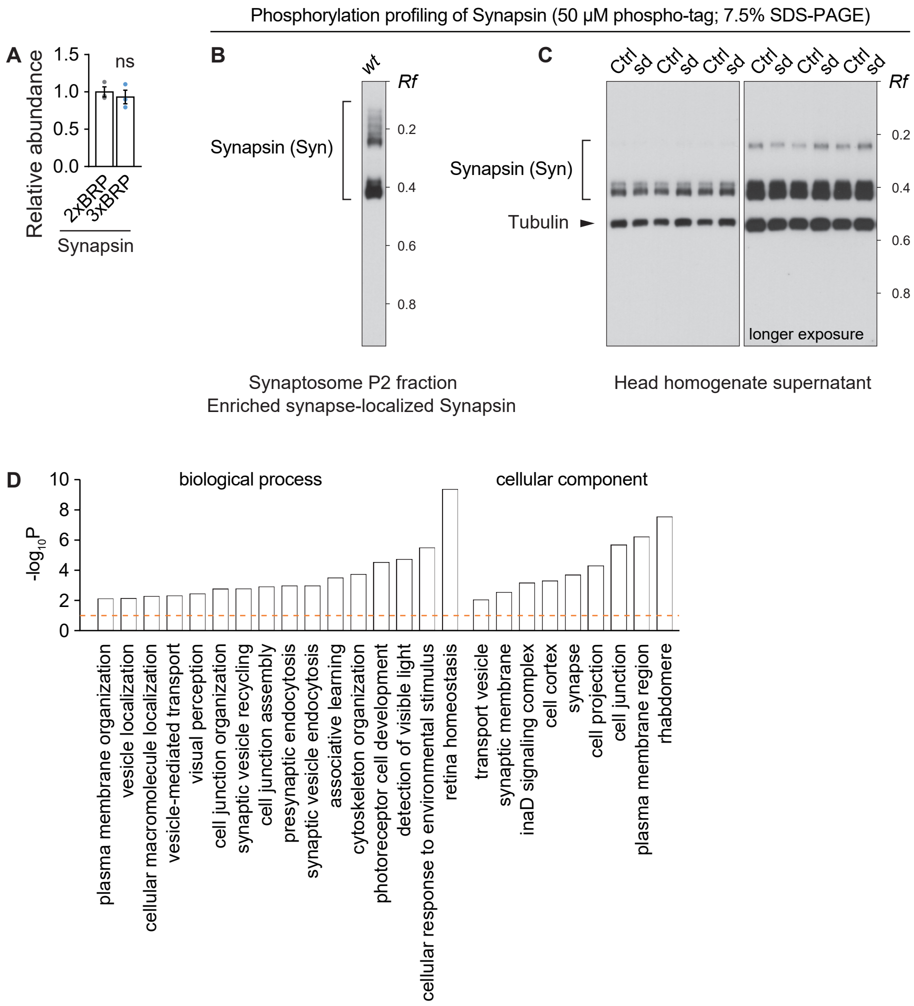

Fig. S3. Phosphorylation profiling of Synapsin in the head homogenate supernatant of sleep-deprived animals.

(**A**) Synaptosomal protein abundance of Synapsin revealed by proteomic analysis. Mann-Whitney test. n = 3.

(**B** and **C**) Direction comparison of phosphorylation profiling of Synapsin between synaptosome P2 fraction (**B**) and head homogenate (**C**). Heavily phosphorylated Synapsin can be observed in synaptosome P2 fraction samples assessed by phospho-tag SDS-PAGE followed by immunoblotting (**B**). Head homogenate samples for either rested *wt* or sleep-deprived *wt* did not show similar pattern (three independent replicates are shown) (**C**).

(**D**) Enriched GO terms in the biological process and cellular component categories of hypophosphorylated proteins in the synaptosome P2 fraction.

ns, not significant. Error bars: mean ± SEM.

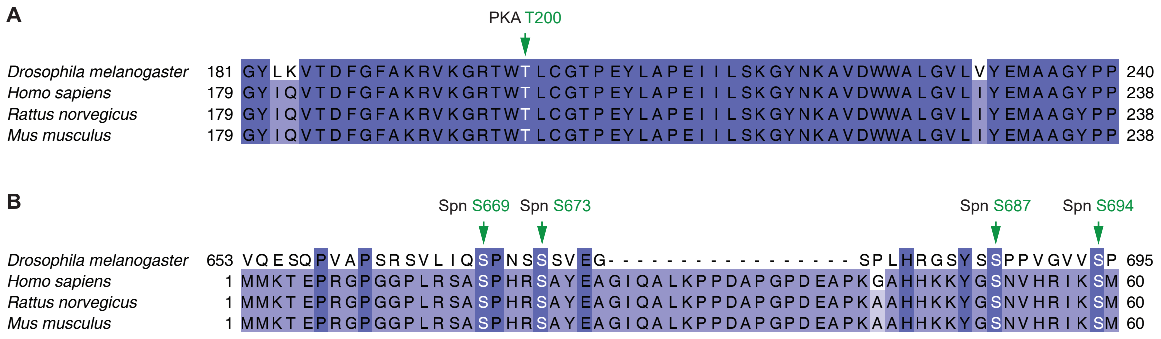

Fig. S4. Evolutionarily conserved phosphorylation sites of Protein Kinase A and Spinophilin.

(**A**) Sequence alignment demonstrating the conserved phospho-site T200 of Protein Kinase A (PKA).

(**B**) Sequence alignment demonstrating 4 conserved phospho-sites of Spinophilin (Spn) including S669, S673, S687 and S694.

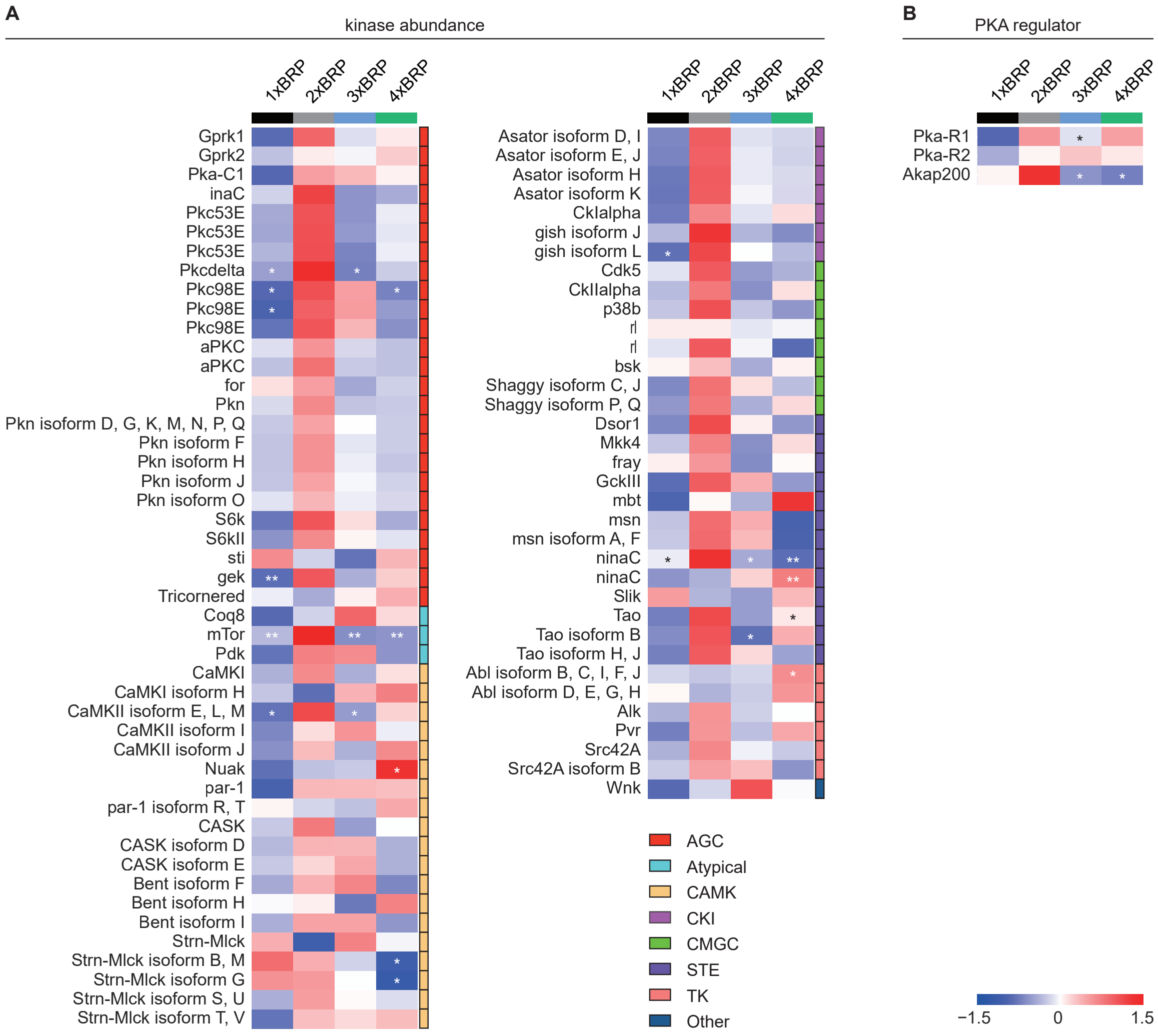

Fig. S5. Protein abundances of detected kinases and PKA regulators.

(**A**) Protein abundances of detected kinases (**A**) and PKA regulators (**B**). The heat maps show the normalized Z-score.

**p*< 0.05; ***p*< 0.01; ****p*< 0.001.

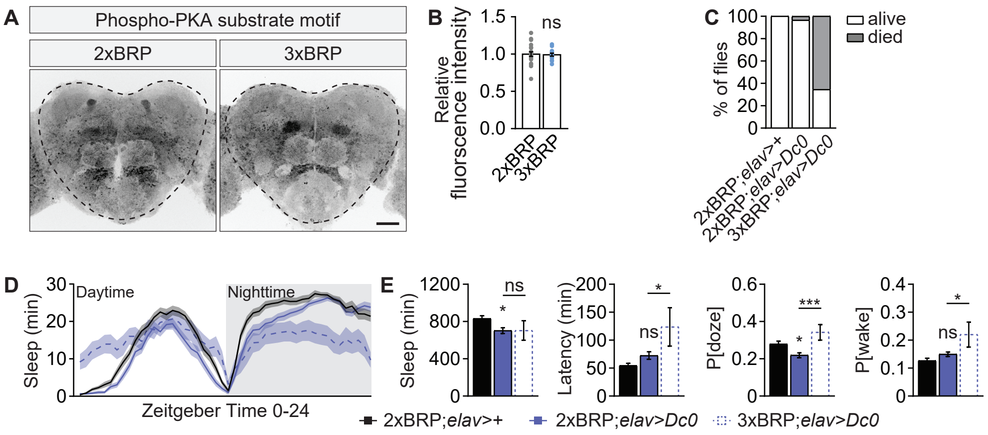

Fig. S6. Evolutionarily conserved phosphorylation sites of Protein Kinase A and Spinophilin.

(**A** and **B**) Representative images (**A**) and statistics (**B**) of whole-mount brain immunostaining against PKA target phosphorylation motifs for 2xBRP *wt* control and 3xBRP flies. Scale bar: 50 µm. Student t test. n = 6-12.

(**C**) Survival of *elav-Gal4* control flies in 2xBRP background, and flies with *elav-Gal4*-driven *Dc0* overexpression in either 2xBRP or 3xBRP background within 5 days' sleep measurements. n = 29-32.

(**D** and **E**) Sleep profile of *elav-Gal4* control flies in 2xBRP background, and flies with *elav-Gal4*-driven *Dc0* overexpression in either 2xBRP or 3xBRP background averaged from measurements over 2–4 days, including sleep curves plotted in 30-min bins (**D**), daily sleep amount, sleep latency at ZT12, P[doze] and P[wake] (**E**). n = 28-32 for 2xBRP;*elav*>+ and 2xBRP;*elav>Dc0*, n = 10 for 3xBRP;*elav>Dc0*.

**p*< 0.05; ****p*< 0.001; ns, not significant. Error bars: mean ± SEM.

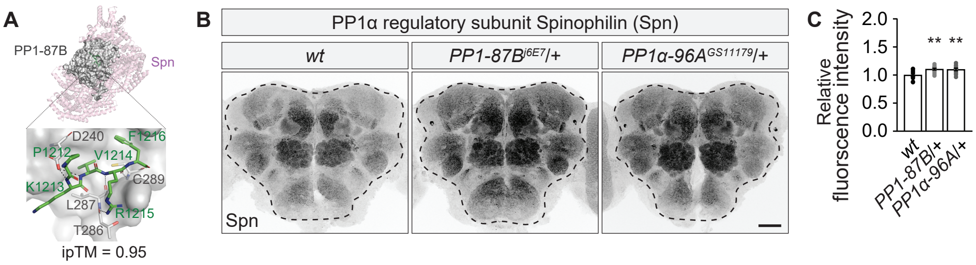

Fig. S7. Predicted interaction between PP1-87B and Spinophilin.

(**A**) AlphaFold3 protein structural prediction for the interaction between Spn and PP1-87B.

(**B** and **C**) Representative images (**B**) and statistics (**C**) of whole-mount brain immunostaining against Spn for heterozygous mutants of PP1-87B and PP1α-96A. Scale bar: 50 μm. One-way ANOVA with Tukey's post hoc tests. n = 12-13

***p*< 0.01; ns, not significant. Error bars: mean ± SEM.

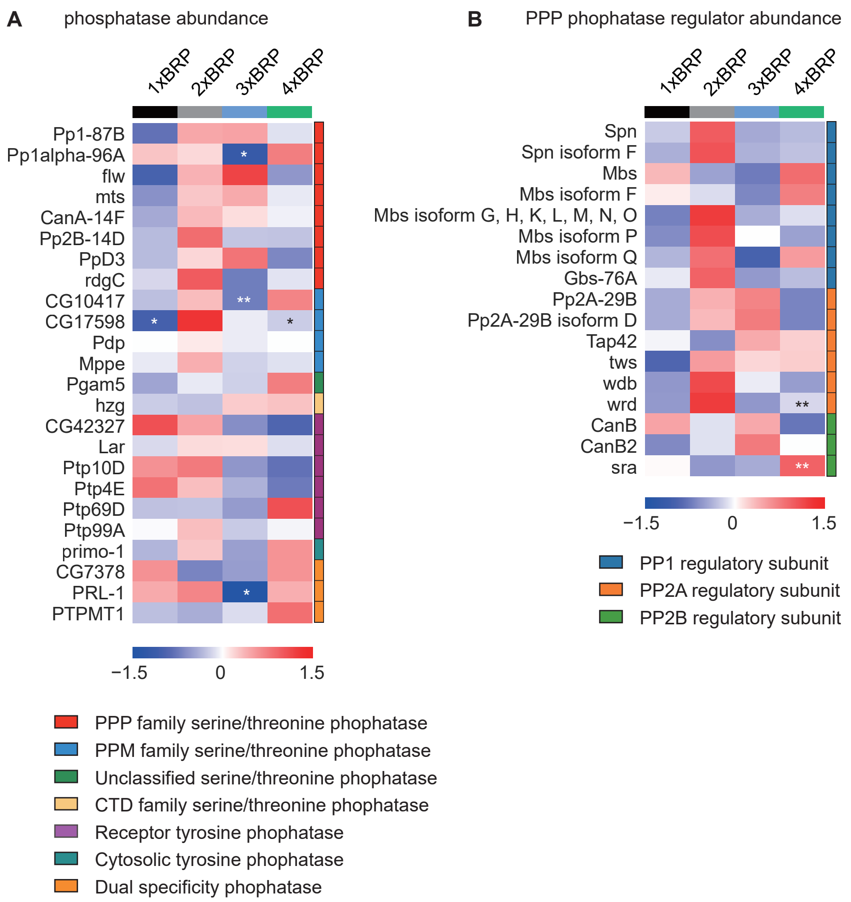

Fig. S8. Protein abundances of detected phosphatases and phosphatase regulators.

(**A**) Protein abundances of detected phosphatases (**A**) and phosphatase regulators (**B**). The heat maps show the normalized Z-score.

**p*< 0.05; ***p*< 0.01; ****p*< 0.001.

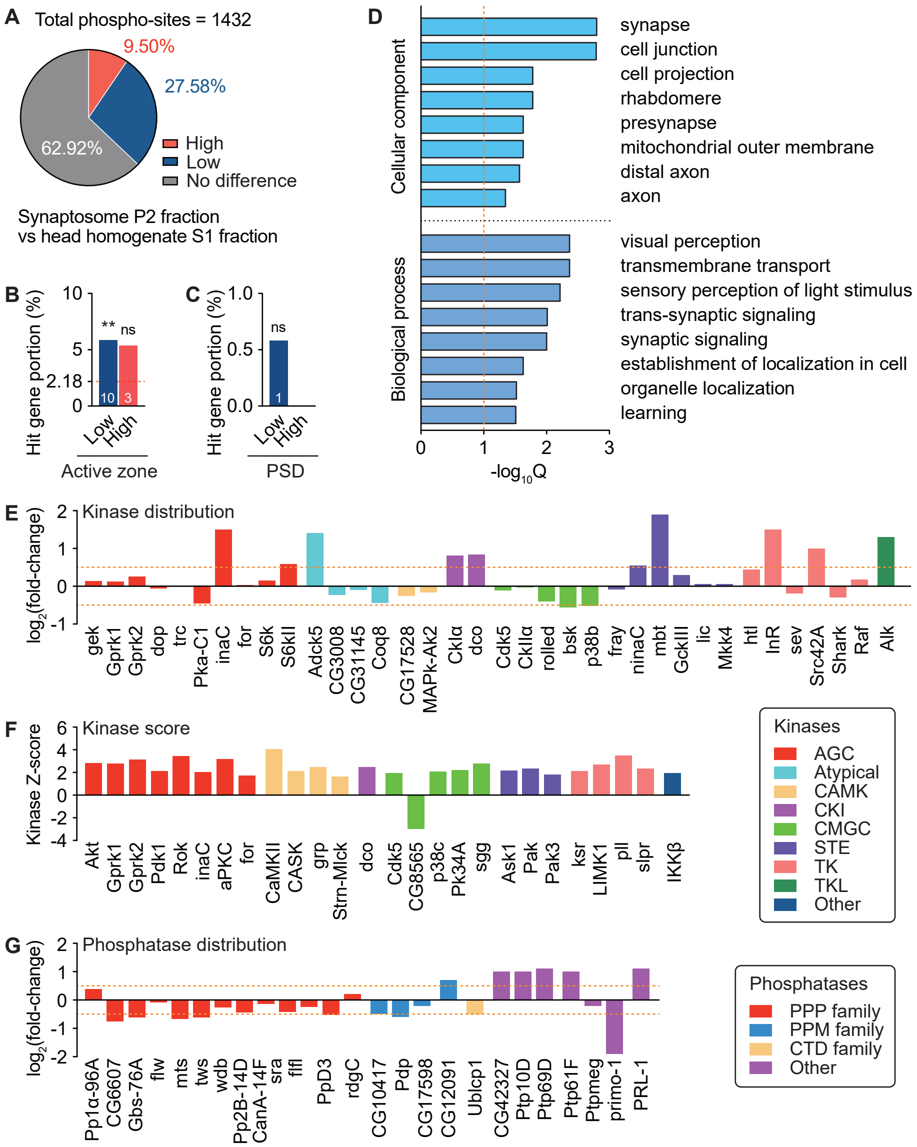

Fig. S9. Phospho-protein distribution across subcellular fractions.

(**A**) Distribution of phospho-peptides with higher or lower expression levels in the synaptosome P2 fraction compared to the head homogenate supernatant S1 fraction.

(**B** and **C**) Percentages of active zone and postsynaptic density (PSD)-related proteins with either higher or lower phosphorylation levels in the P2 fraction compared to the S1 fraction. Fisher's exact test, two-sided.

(**D**) Enriched GO terms in the cellular component and biological process categories of higher and lower phospho-peptides in the P2 fraction compared to the S1 fraction.

(**E**) Expression distribution of detected kinases in P2 and S1 fractions. Bars represent the $\log_2$(fold-change) in kinase abundance when comparing the P2 fraction to the S1 fraction.

(**F**) Predicted kinase activities in P2 and S1 fractions. Bars represent the kinase z-score for each kinase when comparing P2 fraction to the S1 fraction.

(**G**) Expression distribution of detected phosphatases in P2 and S1 fractions. Bars represent the log_2(fold-change) in phosphatase abundance when comparing P2 fraction to the S1 fraction.

** $p < 0.01$; ns, not significant.

References

1. H. Depner, J. Lutzkendorf, H. A. Babkir, S. J. Sigrist, M. G. Holt, Differential centrifugation-based biochemical fractionation of the Drosophila adult CNS. *Nat Protoc* **9**, 2796-2808 (2014).

2. J. M. Burkhart, C. Schumbrutzki, S. Wortelkamp, A. Sickmann, R. P. Zahedi, Systematic and quantitative comparison of digest efficiency and specificity reveals the impact of trypsin quality on MS-based proteomics. *Journal of Proteomics* **75**, 1454-1462 (2012).

3. S. A. Cohen, D. P. Michaud, Synthesis of a Fluorescent Derivatizing Reagent, 6-Aminoquinolyl-N-Hydroxysuccinimidyl Carbamate, and Its Application for the Analysis of Hydrolysate Amino Acids via High-Performance Liquid Chromatography. *Analytical Biochemistry* **211**, 279-287 (1993).

4. N. Shindo *et al.*, Separation of 18 6-Aminoquinolyl-carbamyl-Amino Acids by Ion-Pair Chromatography. *Analytical Biochemistry* **249**, 79-82 (1997).

5. J. V. Olsen *et al.*, Parts per Million Mass Accuracy on an Orbitrap Mass Spectrometer via Lock Mass Injection into a C-trap. *Molecular & Cellular Proteomics* **4**, 2010-2021 (2005).

6. M. C. Sims *et al.*, Novel manifestations of immune dysregulation and granule defects in gray platelet syndrome. *Blood* **136**, 1956-1967 (2020).

7. C. Dickhut, S. Radau, R. P. Zahedi, "Fast, Efficient, and Quality-Controlled Phosphopeptide Enrichment from Minute Sample Amounts Using Titanium Dioxide" in Shotgun Proteomics: Methods and Protocols*,* D. Martins-de-Souza, Ed. (Springer New York, New York, NY, 2014), 10.1007/978-1-4939-0685-7_28, pp. 417-430.

8. O. Pagel, L. Kollipara, A. Sickmann, "Tandem Mass Tags for Comparative and Discovery Proteomics" in Quantitative Methods in Proteomics*,* K. Marcus, M. Eisenacher, B. Sitek, Eds. (Springer US, New York, NY, 2021), 10.1007/978-1-0716-1024-4_9, pp. 117-131.

9. T. Taus *et al.*, Universal and Confident Phosphorylation Site Localization Using phosphoRS. *Journal of Proteome Research* **10**, 5354-5362 (2011).

10. L. Kall, J. D. Canterbury, J. Weston, W. S. Noble, M. J. MacCoss, Semi-supervised learning for peptide identification from shotgun proteomics datasets. *Nat Methods* **4**, 923-925 (2007).

11. G. Shema *et al.*, Simple, scalable, and ultrasensitive tip-based identification of protease substrates. *Molecular & Cellular Proteomics* **17**, 826 (2018).

12. C. Wang *et al.*, GPS 5.0: An Update on the Prediction of Kinase-Specific Phosphorylation Sites in Proteins. *Genomics, Proteomics & Bioinformatics* **18**, 72-80 (2020).

13. P. Casado *et al.*, Kinase-substrate enrichment analysis provides insights into the heterogeneity of signaling pathway activation in leukemia cells. *Sci Signal* **6**, rs6 (2013).

14. D. D. Wiredja, M. Koyuturk, M. R. Chance, The KSEA App: a web-based tool for kinase activity inference from quantitative phosphoproteomics. *Bioinformatics* **33**, 3489-3491 (2017).

15. Z. Wang *et al.*, Quantitative phosphoproteomic analysis of the molecular substrates of sleep need. *Nature* **558**, 435-439 (2018).

16. S. Pounds, C. Cheng, Robust estimation of the false discovery rate. *Bioinformatics* **22**, 1979-1987 (2006).

17. J. Abramson *et al.*, Accurate structure prediction of biomolecular interactions with AlphaFold 3. *Nature* **630**, 493-500 (2024).
